# The Prolyl Isomerase Pin1 Promotes the Herpesvirus-Induced Phosphorylation-Dependent Disassembly of the Nuclear Lamina Required for Nucleocytoplasmic Egress

**DOI:** 10.1371/journal.ppat.1005825

**Published:** 2016-08-24

**Authors:** Jens Milbradt, Corina Hutterer, Hanife Bahsi, Sabrina Wagner, Eric Sonntag, Anselm H. C. Horn, Benedikt B. Kaufer, Yasuko Mori, Heinrich Sticht, Torgils Fossen, Manfred Marschall

**Affiliations:** 1 Institute for Clinical and Molecular Virology, Friedrich-Alexander University of Erlangen-Nürnberg, Erlangen, Germany; 2 Division of Bioinformatics, Institute of Biochemistry, Friedrich-Alexander University of Erlangen-Nürnberg, Erlangen, Germany; 3 Institute for Virology, Freie Universität Berlin, Berlin, Germany; 4 Division of Clinical Virology, Kobe University Graduate School of Medicine, Kobe, Japan; 5 Department of Chemistry, University of Bergen, Bergen, Norway; Louisiana State University Health Sciences Center, UNITED STATES

## Abstract

The nuclear lamina lines the inner nuclear membrane providing a structural framework for the nucleus. Cellular processes, such as nuclear envelope breakdown during mitosis or nuclear export of large ribonucleoprotein complexes, are functionally linked to the disassembly of the nuclear lamina. In general, lamina disassembly is mediated by phosphorylation, but the precise molecular mechanism is still not completely understood. Recently, we suggested a novel mechanism for lamina disassembly during the nuclear egress of herpesviral capsids which involves the cellular isomerase Pin1. In this study, we focused on mechanistic details of herpesviral nuclear replication to demonstrate the general importance of Pin1 for lamina disassembly. In particular, Ser22-specific lamin phosphorylation consistently generates a Pin1-binding motif in cells infected with human and animal alpha-, beta-, and gammaherpesviruses. Using nuclear magnetic resonance spectroscopy, we showed that binding of Pin1 to a synthetic lamin peptide induces its *cis/trans* isomerization *in vitro*. A detailed bioinformatic evaluation strongly suggests that this structural conversion induces large-scale secondary structural changes in the lamin N-terminus. Thus, we concluded that a Pin1-induced conformational change of lamins may represent the molecular trigger responsible for lamina disassembly. Consistent with this concept, pharmacological inhibition of Pin1 activity blocked lamina disassembly in herpesvirus-infected fibroblasts and consequently impaired virus replication. In addition, a phospho-mimetic Ser22Glu lamin mutant was still able to form a regular lamina structure and overexpression of a Ser22-phosphorylating kinase did not induce lamina disassembly in Pin1 knockout cells. Intriguingly, this was observed in absence of herpesvirus infection proposing a broader importance of Pin1 for lamina constitution. Thus, our results suggest a functional model of similar events leading to disassembly of the nuclear lamina in response to herpesviral or inherent cellular stimuli. In essence, Pin1 represents a regulatory effector of lamina disassembly that promotes the nuclear pore-independent egress of herpesviral capsids.

## Author Summary

Viruses often adopt preexisting cellular pathways to promote their own replication. In this regard, the recently discovered alternative mechanism for the nuclear export of large messenger ribonucleoprotein (mRNP) complexes is particularly noteworthy. This process is mechanistically similar to the nuclear egress of herpesviruses, which appear to utilize cellular pathways and effectors to release assembled capsids from the host nucleus. While vesicle formation and scission events at nuclear membranes are now increasingly understood in greater detail, the precise mechanism of the preceding disassembly of the nuclear lamina still awaits a defined molecular characterization. Here, we used herpesviruses in their property to induce a nucleocytoplasmic viral capsid export for our investigation of nuclear lamina disassembly. We identified a mechanism that promotes lamina disassembly by a conformational change of lamins, mediated by the cellular isomerase Pin1 in a phosphorylation-dependent manner. Intriguingly, Pin1 appeared to control the rearrangement of phosphorylated lamins and their transient displacement from the nuclear lamina. Our study suggests that Pin1 functions as a major regulatory effector of lamina disassembly and thus determines the nuclear egress pathway of herpesviruses.

## Introduction

The nuclear envelope represents a physical barrier separating the nucleus from the cytoplasm. It consists of three distinct elements: nuclear membranes, nuclear pores, and the proteinaceous network of the nuclear lamina. The main constituents of the nuclear lamina are nuclear lamins that belong to type V intermediate filament proteins and are grouped into type A and B. Type A includes lamins A and C (lamin A/C) that are derived from the LMNA gene by alternative splicing [1]. Like all intermediate filaments, lamins are composed of a central alpha-helical coiled-coil rod domain flanked by globular head (N-terminal) and tail (C-terminal) domains. Parallel lamin dimers are formed by coiled-coil interactions of the rod domains of two lamin monomers. The lamin dimers form polar head-to-tail polymers, and several polymers associate side by side into lamin filaments [2].

Reversible disassembly of the nuclear lamina was described for several cellular processes including mitosis and nuclear export of large messenger ribonucleoprotein (mRNP) complexes. During open mitosis of higher eukaryotes, lamina disassembly starts in early prophase, and reassembly of the nuclear lamina is completed in telophase [3]. Recent work by Speese *et al*. [4] demonstrated an alternative route for nuclear mRNP export besides translocation of mRNP complexes through nuclear pores. In particular, the alternative export process is based on membrane budding of mRNP complexes at the nuclear envelope which requires local disassembly of the nuclear lamina. Intriguingly, this pathway closely resembles nuclear egress of herpesvirus capsids [5].

Herpesviruses are large, enveloped viruses with linear double-stranded DNA genomes. They are divided into alpha-, beta-, and gammaherpesvirus subfamilies based on their cell tropism, productive replication, latency, and genome sequence. Primary infections are followed by lifelong persistence in their hosts. However, viral pathogenesis and clinical manifestations can differ substantially between individual herpesviruses [6]. Synthesis of viral genomic DNA and assembly of viral capsids occur in the nucleus. Upon packaging of the genome, capsids are translocated into the cytoplasm. This multistage process is termed nuclear egress and is crucial for herpesviral replication [7]. Due to their large size, herpesviral capsids cannot be transported through nuclear pores. Instead, nucleocytoplasmic transport of viral capsids is mediated by budding through nuclear membranes. Notably, access of viral capsids to the inner nuclear membrane (INM) is impeded by the nuclear lamina. A large body of evidence established that nuclear egress of herpesviral capsids is facilitated by local disassembly of the nuclear lamina [reviewed in 8].

Site-specific phosphorylation of lamin A/C at Ser22 and Ser392, which is mainly mediated by cyclin-dependent kinase 1 (CDK1), promotes the transient disassembly of the nuclear lamina during mitosis [9,10]. However, it seems that phosphorylation of one of these ‘mitotic sites’, namely Ser22, is sufficient to induce disassembly of at least lamin A/C [11]. Besides Ser22 and Ser392, mitotic phosphorylation of further serine residues by CDK1, such as Ser404 and Ser406, was shown to be associated with increased depolymerization of lamin filaments [12]. It is generally accepted that herpesvirus-induced lamina disassembly during nuclear egress is based on a similar phosphorylation-dependent process [13–15].

Importantly, our studies on nuclear egress of the human cytomegalovirus (HCMV) suggested that the cellular peptidyl-prolyl *cis/trans* isomerase (PPIase) Pin1 is involved in lamina disassembly during herpesvirus infection [16]. Pin1 is a nuclear PPIase that induces conformational changes in its substrates by isomerization of phosphorylated Ser/Thr-Pro bonds [17]. Notably, we recognized that Ser22-specific phosphorylation, mediated by the viral protein kinase pUL97 during HCMV infection, generates a Pin1-binding motif in lamin A/C. Moreover, we demonstrated coprecipitation of lamin A/C by a Pin1 antibody from HCMV-infected cell lysates and translocation of Pin1 to the nuclear periphery of HCMV-infected cells [16].

In this study, we investigated the role of Pin1 during herpesviral nuclear egress and, particularly, its importance for lamina disassembly in general. Phosphorylation of Ser22 of lamin A/C consistently generates a Pin1-binding motif in cells infected with human and animal alpha-, beta-, and gammaherpesviruses. Using nuclear magnetic resonance (NMR) spectroscopy, we demonstrated that binding of human Pin1 to a synthetic lamin peptide induces its *cis/trans* isomerization *in vitro*. A detailed bioinformatic evaluation strongly suggests that this structural conversion induces larger scale secondary structural changes in the lamin N-terminus. Thus, we propose that a Pin1-induced conformational change of lamins may result in the disassembly of the nuclear lamina. Consistent with this concept, we observed that a phospho-mimetic lamin A mutant still forms a regular lamina structure and that overexpression of a lamin-phosphorylating kinase is not sufficient to induce lamina disassembly in Pin1 knockout (KO) cells. Furthermore, pharmacological inhibition of Pin1 interfered with the efficient lamina disassembly in herpesvirus-infected human fibroblasts, and consequently, resulted in decreased virus replication. Pin1 inhibition also causes association of phosphorylated lamins with the nuclear lamina, which would become dispersed throughout the nucleus without inhibition. Strikingly, we observed this phenomenon not only in herpesvirus-infected cells but also in uninfected cells. Our results therefore suggest that a Pin1-induced conformational change of lamins generally facilitates disassembly of the nuclear lamina.

## Results

### Ser22-specific phosphorylation of lamin A/C is conserved during alpha-, beta- and gammaherpesvirus replication

**Fig 1:**
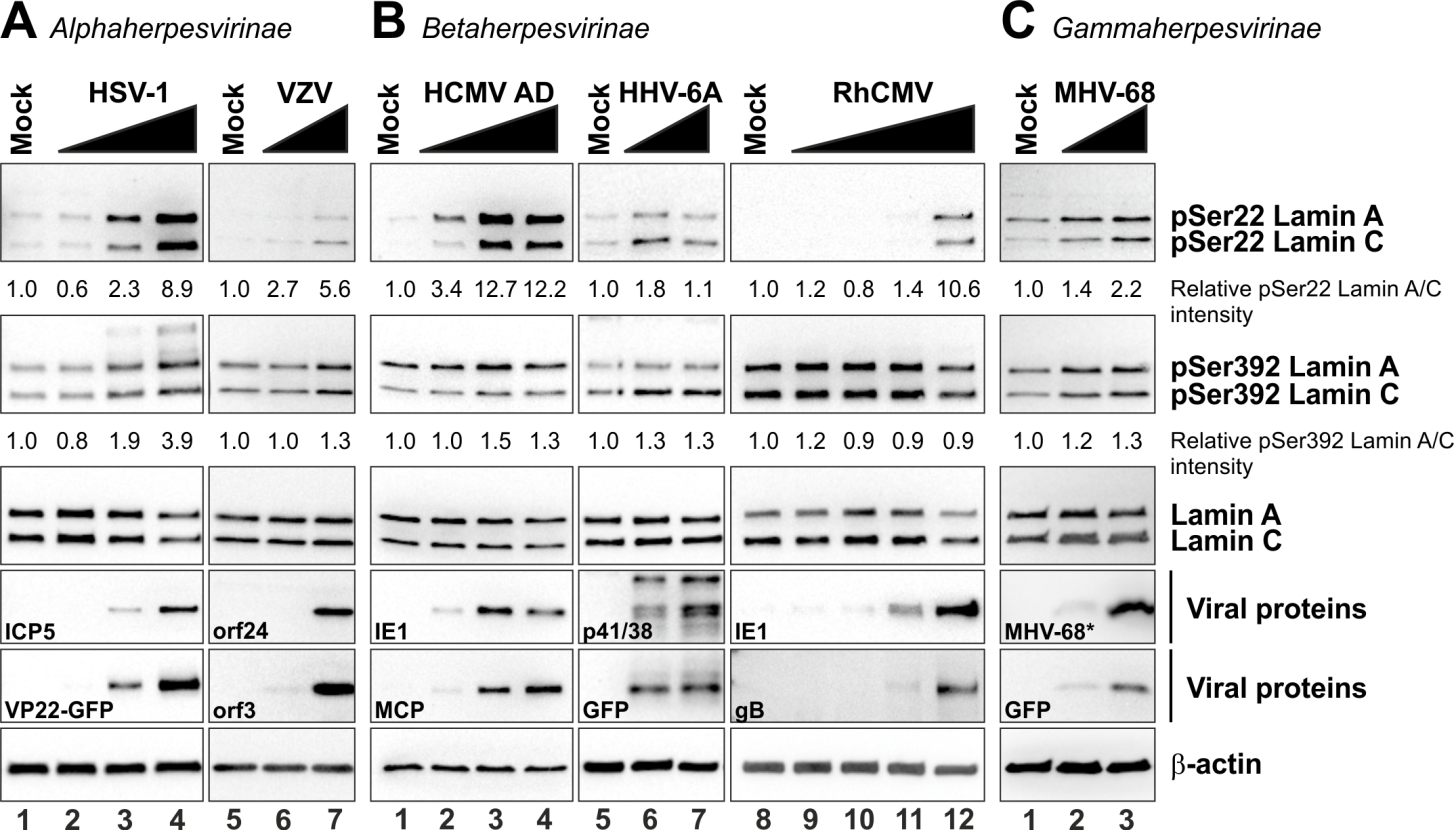
Site-specific phosphorylation of lamin A/C at Ser22 and Ser392 in herpesvirus-infected primary fibroblasts analysed by western blot. HFFs were infected with different herpesviruses belonging to subfamily alpha (A), beta (B), and gamma (C). For HSV-1, HCMV, RhCMV, and MHV-68 infections, increasing MOIs were applied in a range between approx. 0.1–1. Cells were lysed at 24 hpi (HSV-1) or 72 hpi (HCMV, RhCMV, and MHV-68). Virus-positive carrier cells were cocultivated in serial dilutions with host HFFs for VZV and HHV-6A infections, producing ~60% and ~30% of infected cells, respectively. Cells were lysed at 72 h post-cocultivation. In all cases, total lysates were subjected to standard Western blot analysis for detection of lamin A/C phosphorylated at Ser22 (pSer22) or Ser392 (pSer392) (upper two panels), total lamin A/C (third panels), viral marker proteins (fourth and fifth panels), and loading control β-actin (lower panels). *, detection of a viral 17 kDa protein using a polyspecific MHV-68 post-infection murine antiserum. Ser22 and Ser392 phosphorylation signal intensities were quantified and related to the lamin A/C and β-actin signals by densitometry using AIDA image analyser.

**Fig 2:**
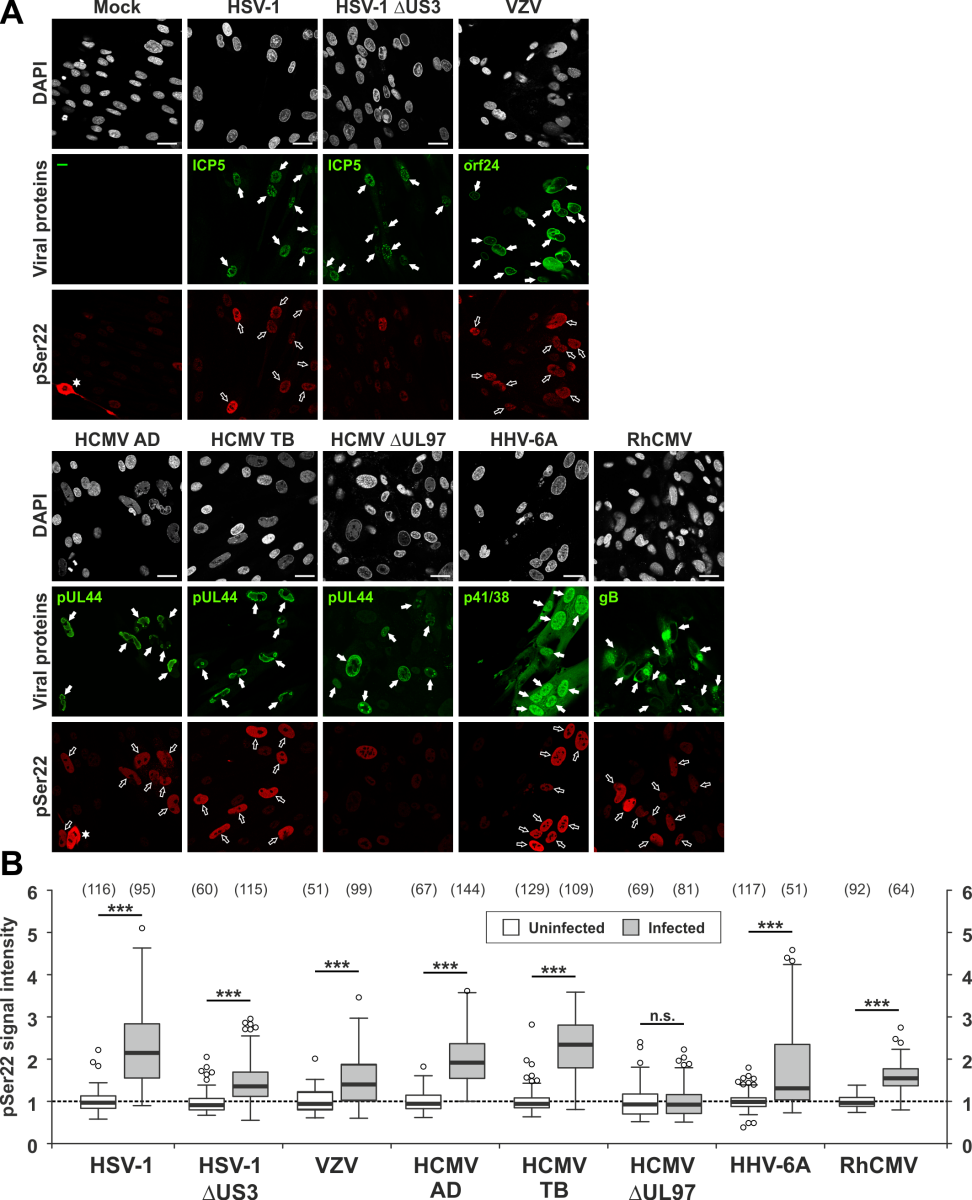
Ser22-specific phosphorylation of lamin A/C in herpesvirus-infected primary fibroblasts analysed by confocal imaging. (A) HFFs were infected with different herpesviruses or remained uninfected (mock) as indicated. Cells were fixed at 24 hpi (HSV-1 and HSV-1 ΔUS3) or 72 hpi (VZV, HCMV AD, HCMV TB, HCMV ΔUL97, HHV-6A, and RhCMV) followed by immunofluorescence analysis using phospho-specific antibodies to detect lamin A/C phosphorylated at Ser22 in red. Staining of viral proteins or the green fluorescent protein (GFP) served as viral markers in green. Cell nuclei were counterstained with DAPI (4’,6-diamidino-2-phenylindole). Samples were analysed by confocal microscopy and a representative image of the focal plane is depicted for each setting. *Filled arrows*, nuclei of virus-positive cells; *open arrows*, nuclei of virus-positive cells showing increased Ser22 phosphorylation compared to virus-negative cells; *scale bars*, 30 μm. (B) Median intracellular intensities of lamin A/C phosphorylation. pSer22 signals were determined for infected (white boxes) and surrounding uninfected cells (grey-shaded boxes) as maximum projections of confocal z-series. One representative experiment out of three is depicted for each virus presenting the values of site-specific phosphorylation as box plots. Note, Table 2 contains the mean values ± standard deviation of three independent experiments. Centre lines show the medians with box limits indicating the 25th and 75th percentiles as determined by R software. Whiskers extend 1.5 times the interquartile range from the 25th and 75th percentiles, outliers are represented by circles, and the number of evaluated cells is depicted above each box in brackets. Statistical significance was determined by Student’s t-test (*, P < 0.05; **, P < 0.01; ***, P < 0.001; *n*.*s*., not significant, P ≥ 0.05).

Site-specific phosphorylation of nuclear lamins has been considered a trigger for lamina disassembly during both mitosis and herpesvirus infection [8,14,18]. In this study, we analysed phosphorylation of ‘mitotic sites’ in lamin A/C during replication of alpha-, beta-, and gammaherpesviruses (Figs 1, 2 and Table 1). First, to further investigate the importance of site-specific phosphorylation during HCMV replication, we infected primary human foreskin fibroblasts (HFFs) with HCMV laboratory strain AD169 (HCMV AD), harvested the cells at 72 hours post-infection (hpi), and performed Western blot analysis using phosphorylation-specific antibodies. While the expression level of lamin A/C remained constant (Fig 1B, lanes 1–4, third panel), a multiplicity of infection (MOI)-dependent increase of Ser22 phosphorylation was detected in HCMV-infected cells compared to the uninfected control (mock; Fig 1B, lanes 1–4, upper panel). In contrast, phosphorylation of Ser392 was detectable, but not increased in HCMV-infected cells (Fig 1B, lanes 1–4, second panel). Notably, the fact that Ser22 and Ser392 are generally phosphorylated in HCMV-infected cells was previously described by Hamirally *et al*. [19]. However, our data indicate that only Ser22 phosphorylation is specifically induced during HCMV replication whereas Ser392 phosphorylation levels are not considerably elevated in HCMV-infected cells.

**Table 1:** Virus strains and recombinant viruses used in this study.

Name	HV number	Virus strain	Recombinant protein	Mutation	Sub-family	Natural host
**HSV-1**	HHV-1	166v	VP22-GFP	/	α	Human
**HSV-1 ΔUS3**	HHV-1	R7037	/	ΔUS3	α	Human
**VZV**	HHV-3	Oka	/	/	α	Human
**HCMV AD**	HHV-5	AD169	/	/	β	Human
**HCMV TB**	HHV-5	TB40	pUL32-GFP	/	β	Human
**HCMV GFP**	HHV-5	AD169	GFP	/	β	Human
**HCMV ΔUL97**	HHV-5	AD169	GFP	ΔUL97	β	Human
**HHV-6A**	HHV-6A	U1102	GFP	/	β	Human
**RhCMV**	RhHV-5	68–1	/	/	β	Rhesus macaque
**MHV-68**	MuHV-4	68	GFP	/	γ	Bank vole

*HV*, herpesvirus; *HSV-1*, herpes simplex virus type 1; *VZV*, varicella zoster virus; *HCMV AD*, HCMV laboratory strain AD169; *HCMV TB*, recombinant HCMV strain TB40; *HHV-6A*, human herpesvirus 6A; *RhCMV*, rhesus cytomegalovirus; *MHV-68*, murine herpesvirus 68; *GFP*, green fluorescent protein.

In the next step, we compared phosphorylation levels of Ser22 and Ser392 in cells infected with representatives of the three subfamilies of *Herpesviridae* in addition to HCMV: i.e. three human viruses (HSV-1, VZV, and HHV-6A), one non-human primate virus (RhCMV), and one murine virus (MHV-68). Similarly to HCMV, these viruses have the ability to infect HFFs under cell culture conditions. While HFFs are not susceptible to infection with the human gammaherpesviruses EBV and KSHV, infection with murine MHV-68 was positive in leading to the expression of viral proteins and site-specific lamin phosphorylation (Fig 1C). Intriguingly, Ser22 phosphorylation consistently increased in cells infected with the analysed herpesviruses (Fig 1A–1C, upper panels), while Ser392 was phosphorylated in a virus-specific manner. In particular, a strong increase of Ser392 phosphorylation compared to uninfected cells was detected for HSV-1 (Fig 1A, lanes 1–4, second panel), but no increase for VZV, HHV-6A, RhCMV, and MHV-68 (Fig 1A, lanes 5–7, Fig 1B, lanes 5–12, and Fig 1C, lanes 1–3, second panels). Lamin A/C expression levels remained unaltered for HSV-1, RhCMV, MHV-68, VZV, and HHV-6A (Fig 1A–1C, third panels).

In addition to Western blot analysis, cells were subjected to confocal immunofluorescence microscopy (Fig 2 and S1 Fig). Notably, viral proteins stained as markers for infection are expressed at early (E) or late (L) kinetics: the viral DNA polymerase processivity factors pUL44 and p41 of HCMV and HHV-6A, respectively, and the nuclear egress protein encoded by orf24 of VZV are E gene products; the major capsid protein ICP5 of HSV-1 and glycoprotein B (gB) of RhCMV are L gene products. While nuclear egress is expected to occur at the L phase of viral replication, Western blot kinetics experiments showed that lamin phosphorylation is already markedly increased along the proceeding of the E phase (i.e. ≥ 48 hpi) of HCMV replication (S2 Fig). Lamin A/C and lamin B differ in their ability to remain associated with the INM. Whereas lamin A/C can be found solubilized in the nucleus, lamin B is permanently membrane associated due to post-translational isoprenylation and specific protein interactions with membrane proteins such as the lamin B receptor [20]. We detected dispersed lamin A/C phosphorylation signals in virus-infected cells entirely inside the nucleus by confocal microscopy (Fig 2A and S1A Fig). The localization of phosphorylated lamins in infected cells clearly differed from mitotic cells that showed a wide nucleocytoplasmic pSer22 distribution (Fig 2A, panels Mock and HCMV AD, indicated by asterisks). We quantified signal intensities of lamin A/C phosphorylation in virus-infected cells in comparison to uninfected cells within z-series for individual nuclei with standardized conditions and identical imaging areas (Fig 2B). Staining of viral marker proteins was used to localize infected cells. Importantly, signal intensities of Ser22 phosphorylation were increased in more than 80% of cells infected with HSV-1, HCMV AD, HCMV TB, and RhCMV to approx. 2-fold over uninfected cells (Fig 2B and Table 2). For VZV and HHV-6A, more than 50% of infected cells showed a moderate increase in Ser22 phosphorylation (1.41±0.09- and 1.69±0.20-fold, respectively). In case of HCMV infection, Ser22 phosphorylation was dependent on the viral protein kinase pUL97 as seen with HCMVs AD, TB and ΔUL97 (a recombinant HCMV lacking pUL97 kinase). In particular, infection with HCMV ΔUL97 did not alter Ser22 phosphorylation compared to uninfected cells. In contrast, cells infected with HSV-1 ΔUS3, lacking pUS3 kinase, showed a moderately lower Ser22 phosphorylation efficiency than those infected with parental HSV-1, but still increased compared to uninfected cells (Fig 2B, Table 2, and S1 Dataset). This suggests that Ser22 might also be phosphorylated by the second viral protein kinase, pUL13, or cellular kinases (such as protein kinase C, PKC) in HSV-1-infected cells.

**Table 2:** Summary of site-specific lamin phosphorylation analysed in this study.

Virus	pSer22			pSer392		
	Wb	CLSM		Wb	CLSM	
		Fold change	% of infected cells		Fold change	% of infected cells
**HSV-1**	**strong**	**2.48 ± 0.17**	**80.94 ± 6.58**	**strong**	**2.11 ± 0.42**	**86.23 ± 9.48**
		P < 0.0001	P < 0.0001		P = 0.01	P = 0.0003
**HSV-1 ΔUS3**	n.d.	**1.41 ± 0.06**	**45.94 ± 17.21**	n.d.	**1.33 ± 0.11**	**51.29 ± 9.40**
		P = 0.0003	P = 0.042		P = 0.0055	P = 0.0029
**VZV**	**strong**	**1.41 ± 0.09**	**53.19 ± 5.91**	none	n.d.	n.d.
		P = 0.0014	P < 0.0001			
**HCMV AD**	**strong**	**1.96 ± 0.39**	**89.06 ± 4.36**	weak	0.98 ± 0.03	16.43 ± 2.26
		P = 0.0025	P < 0.0001		P = 0.32	P = 0.9
**HCMV TB**	n.d.	**2.21 ± 0.07**	**84.77 ± 6.57**	n.d.	0.98 ± 0.05	15.27 ± 3.15
		P < 0.0001	P < 0.0001		P = 0.47	P = 0.97
**HCMV ΔUL97**	n.d.	1.03 ± 0.02	12.75 ± 1.83	n.d.	0.62 ± 0.02	1.82 ± 1.60
		P = 0.067	P = 0.15		P < 0.0001	P = 0.0002
**HHV-6A**	weak	**1.69 ± 0.20**	**59.98 ± 2.74**	none	1.08 ± 0.01	17.81 ± 5.14
		P = 0.0039	P < 0.0001		P < 0.0001	P = 0.19
**RhCMV**	**strong**	**1.54 ± 0.03**	**85.61 ± 7.19**	none	0.91 ± 0.05	9.03 ± 4.36
		P < 0.0001	P < 0.0001		P = 0.035	P = 0.13
**MHV-68**	**strong**	n.d.	n.d.	none	n.d.	n.d.
					

Lamin phosphorylation at Ser22 (pSer22) and Ser392 (pSer392) was determined in herpesvirus-infected HFFs by Western blot (Wb) analyses and confocal laser-scanning microscopy (CLSM). A significant increase in lamin phosphorylation in infected cells compared to uninfected control cells is shown in *bold*. Quantitative determination by Wb analysis: *strong*, > 2-fold increase of phosphorylation signals in virus-infected cells compared to mock-infected cells; *weak*, 1.5–2.0-fold increase of phosphorylation signals; *none*, no increase of phosphorylation signals (< 1.5-fold change). Quantitative evaluation by CLSM: *Fold change*, mean signal intensities in infected cells compared to uninfected cells; *% of infected cells*, percentage of lamin phosphorylation-increased infected cells compared to uninfected cells (any increase in signals, mean ± standard deviation). Results are presented as a mean of at least three experiments; statistical sigificance is given by *P values*. *n*.*d*., not determined.

As far as Ser392 phosphorylation is concerned, 86.23±9.48% of HSV-1-infected cells showed an increase in signal intensities (2.11±0.42-fold), whereas infections with betaherpesviruses (i.e. HCMV AD, HCMV TB, HHV-6A, and RhCMV) did not alter Ser392 phosphorylation (S1B Fig, Table 2, and S2 Dataset). Similar to Ser22 phosphorylation, 51.29±9.40 of cells infected with HSV-1 ΔUS3 showed a moderate increase in Ser392 phosphorylation (1.33±0.11-fold). Taken together, Western blot analyses and quantitative confocal microscopy revealed consistently increased Ser22 phosphorylation levels for all viruses used, compared to a virus-specific increase of Ser392 phosphorylation particularly found for HSV-1 (Table 2).

### The cellular PPIase Pin1 promotes efficient herpesviral replication

**Fig 3:**
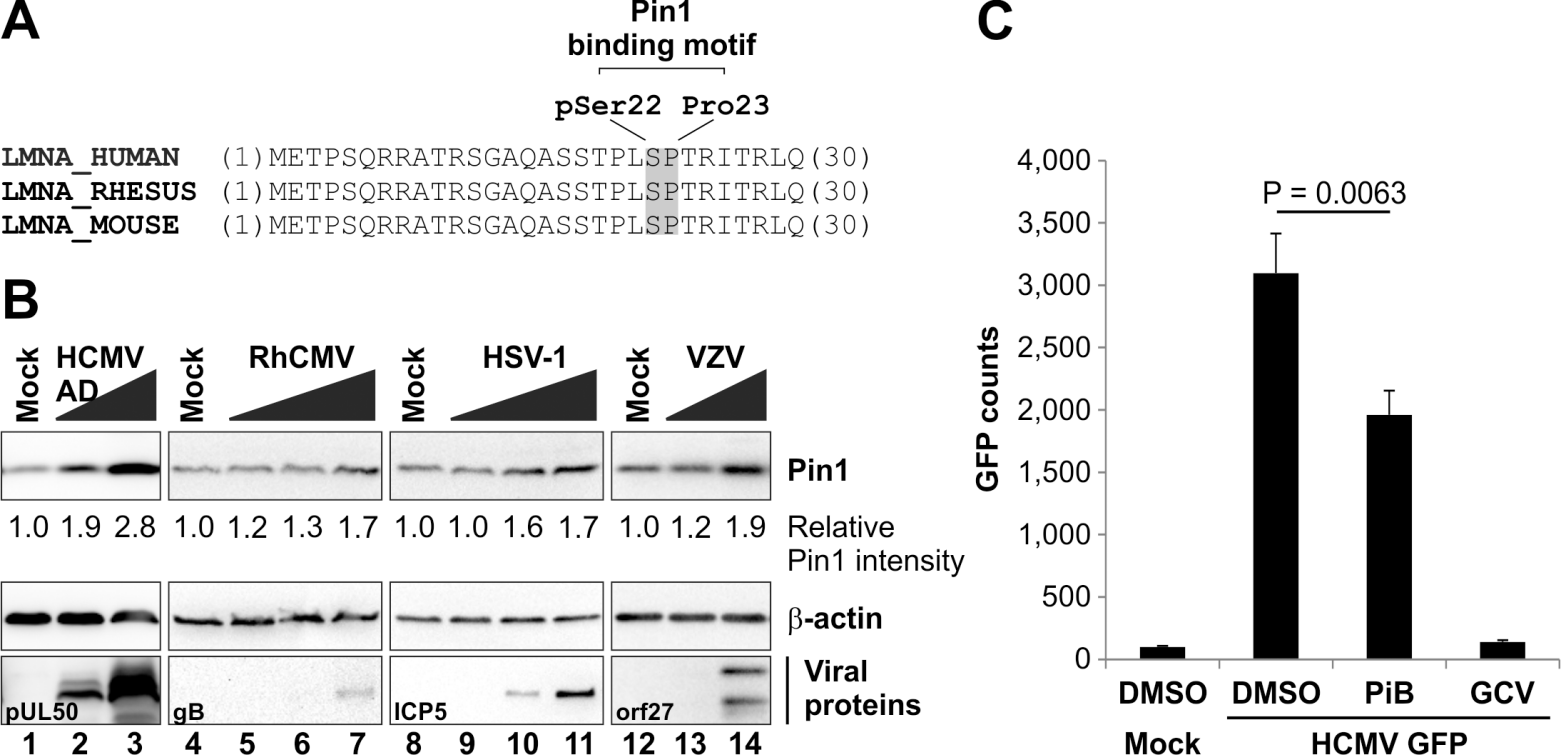
Putative role of Pin1 in herpesviral replication. (A) Conserved Pin1-binding motif in lamin A/C. Amino acid sequences of the lamin A/C precursor from humans (UniProt accession number: P02545), rhesus macaques (F7GLE9), and mice (P48678) were analysed by multiple sequence alignment. Note the 100% conservation of the lamin A/C N-terminus including the Pin1-binding motif. Depicted are the N-terminal 30 amino acids of each sequence. (B) Pin1 upregulation in herpesvirus-infected cells. HFFs were infected with HSV-1, VZV, HCMV, or RhCMV at increasing MOIs. Cells were lysed at 24 hpi (HSV-1) or 72 hpi (VZV, HCMV, and RhCMV). Lysates were subjected to standard Western blot analysis for detection of Pin1 (rabbit mAb-Pin1; upper panels), viral marker proteins (middle panels), and loading control β-actin (lower panels). Pin1 signal intensities were quantified by densitometry using AIDA image analyser. (C) Effect of pharmacological Pin1 inhibition on herpesviral replication efficiency. HFFs were infected with HCMV GFP at a MOI of 0.2 or remained uninfected (mock). The Pin1 inhibitor PiB and the anti-HCMV reference drug ganciclovir (GCV) were added immediately post-infection at 10 μM and 20 μM, respectively. Cells were lysed at 7 days post-infection to perform quantitative GFP fluorometry (n = 3; mean ± standard deviation; statistical significance was determined by Student’s t-test). Potential cytotoxic effects of these compounds in the concentrations used were excluded by microscopic evaluation before cell lysis.

We previously identified Pin1 as a phosphorylation-dependent binding partner of lamin A/C in HCMV-infected cells, suggesting that Pin1 might play a role during herpesvirus-induced lamina disassembly [16]. Analyses of lamin phosphorylation during infection with selected members of the *Herpesviridae* revealed the generation of a common Pin1-binding motif in lamin A/C comprising pSer22 and Pro23. A multiple sequence alignment of LMNA sequences demonstrated conservation of the Pin1-binding motif at least in lamin A/C of humans, rhesus macaques, and mice (Fig 3A). We then analysed Pin1 expression levels in infected cells by Western blot analysis. Since Pin1 expression is activated by the transcription factor E2F [21], which is subject to the control of cell cycle progression [22], HHFs were synchronized at the G0 phase by serum starvation prior to HCMV infection. At 72 hpi, an upregulation of Pin1 was demonstrated in an MOI-dependent manner (Fig 3B, lanes 1–3). An increase in Pin1 levels was also observed for infection with HSV-1, VZV, or RhCMV (Fig 3B, lanes 4–14), but not as efficient as seen for HCMV. The upregulation of Pin1 expression might be explained by the fact that several herpesviruses are able to activate E2F-mediated gene expression by a modulation of the degree of phosphorylation and degradation of the major cell cycle-controlling retinoblastoma protein [23–25].

To determine if Pin1 PPIase activity possesses a general function during herpesviral replication, we inhibited Pin1 PPIase activity by addition of the Pin1 inhibitor PiB. The effect of Pin1 inhibition on herpesviral replication efficiency was analysed using an established HCMV green fluorescent protein (GFP)-based reporter assay at day 7 post-infection [26]. HCMV replication was reduced by the presence of PiB during infection (Fig 3C). Notably, PiB produced a moderate inhibitory effect when compared to the anti-HCMV reference drug ganciclovir (GCV). Pin1 inhibition by PiB was not associated with general cytotoxicity at the concentration used of 10 μM, indicated by microscopic evaluation of cell morphology and cell growth (S3 Fig). Moreover, the data derived from a standard trypan blue exclusion assay demonstrated that PiB-induced cytotoxicity occurred only with drug concentrations of 60 μM and higher (S4A Fig). The total cell numbers (dead and living cells) were not significantly reduced under PiB treatment (S4B Fig). In conclusion, Pin1 expression is regulated in herpesvirus-infected cells and its activity is important for efficient viral replication.

### Pin1 catalyses the prolyl *cis/trans* isomerization of a phosphorylated lamin A/C peptide

**Fig 4:**
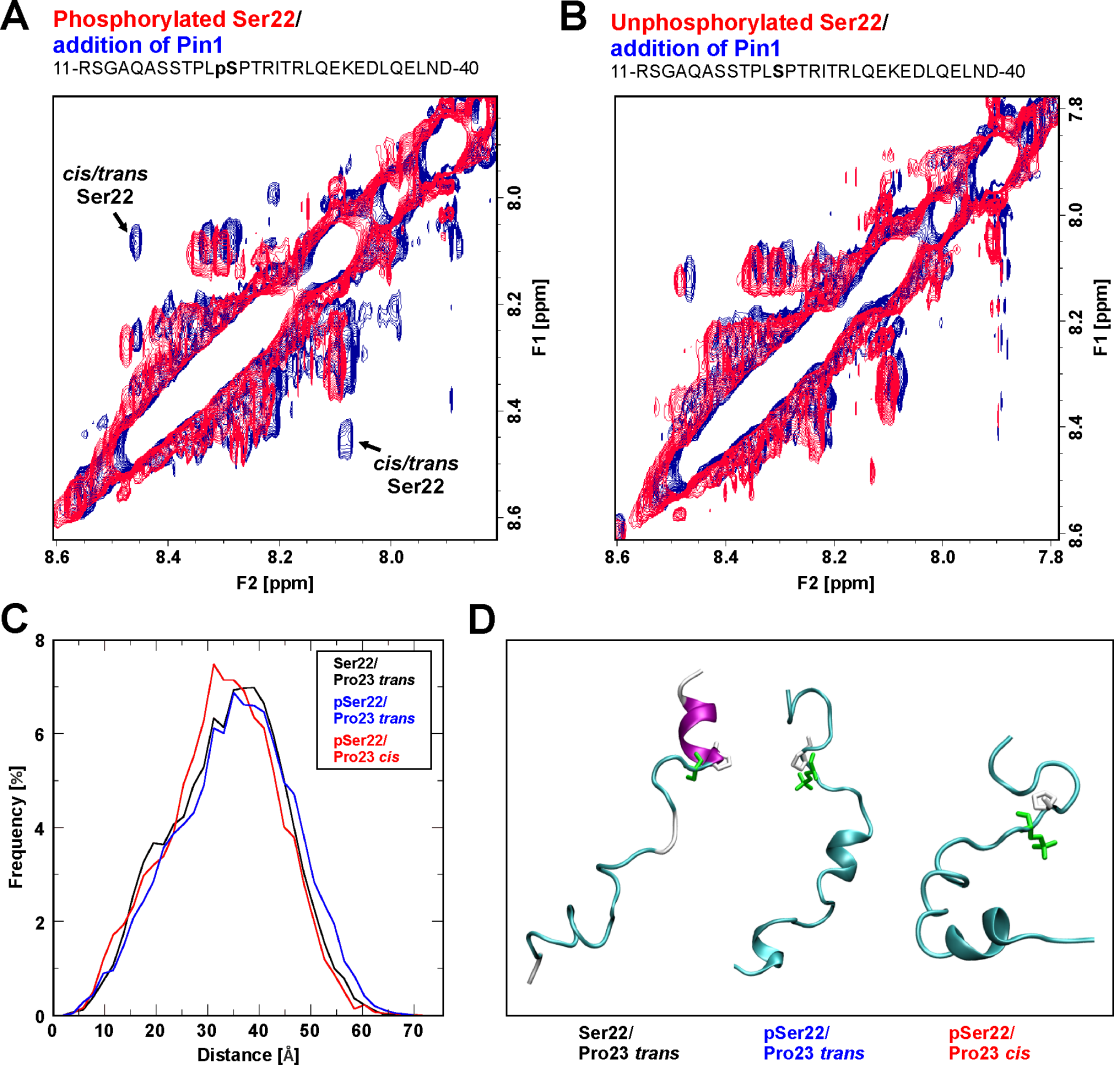
Pin1-induced *cis/trans* isomerization of lamin A/C. (A-B) NMR spectroscopy of lamin A/C peptides in the presence or absence of Pin1. Superimposed expanded HN-HN regions of the 2D ^1^H-^1^H NOESY spectra are depicted for phosphorylated and unphosphorylated versions of a lamin A/C peptide comprising amino acids 11–40. (A) Phosphorylated peptide prior to (red signals) and after addition of Pin1 (blue signals); note the appearance of exchange peaks originating from an enhanced prolyl *cis/trans* interconversion rate after addition of Pin1. (B) Unphosphorylated peptide prior to (red signals) and after addition of Pin1 (blue signals); note that no exchange peaks are observed for the unphosphorylated peptide. (C-D) Molecular dynamics (MD) simulation of lamin A/C peptides. (C) A histogram plot of the end-to-end distances for the lamin A/C (1–30) peptides that differ in the phosphorylation state of Ser22 and the isomerization state of Pro23. (D) Representative snapshots from the different MD simulations indicating the most populated conformation of the Ser22/Pro23 *trans* (left), pSer22/Pro23 *trans* (middle), and pSer22/Pro23 *cis* (right) peptide. Ser22 (green) and Pro23 (white) are highlighted as stick presentations.

Pin1 is a PPIase that isomerizes specific phosphorylated Ser/Thr-Pro motifs resulting in conformational changes in target proteins [17,27]. On this basis we postulated that herpesvirus-mediated and mitotic Ser22 phosphorylation might trigger a Pin1-induced conformational change in lamin A/C. At present, there is no technique available to study prolyl *cis/trans* isomerization at atomic resolution *in vivo*. However, nuclear magnetic resonance (NMR) spectroscopy has been proven as a suitable method to provide information about PPIase interaction [28], in particular for Pin1 activity directed to phosphorylated Pro-containing peptides at atomic resolution [29]. Moreover, we have previously demonstrated that enzymatic catalysis of prolyl *cis/trans* isomerization observed for domain peptides are also valid for the full length protein [30]. In order to assign the ^1^H chemical shifts of phosphorylated or unphosphorylated lamin A/C peptides (comprising amino acids 11 to 40), catalytic amounts of Pin1 were added to the peptide solution and data were then recorded in a complete series of NMR experiments. In the presence of Pin1, exchange peaks between related NH signals from Ser22 of the *cis* Pro23 and *trans* Pro23 isomers of phosphorylated lamin A/C peptides were observed (Fig 4A). In contrast, no exchange peaks were observed after addition of Pin1 to the solution of the analogous unphosphorylated lamin A/C peptide (Fig 4B). This result was not unexpected since phosphorylated Ser and Thr preceding Pro in this type IV WW binding motif have hitherto been recognized as substrates for Pin1 [27,31]. To confirm the specificity of interaction between Pin1 and the Ser22-phosphorylated lamin A/C peptide, the known Pin1 inhibitor juglone was applied [32]. The NOESY NMR spectrum of the phosphorylated lamin A/C peptide revealed that after the sequential addition of Pin1 and juglone, the prolyl *cis/trans* exchange peaks disappeared and the NMR spectra closely resembled those of the untreated phosphorylated peptide (S5 Fig). Thus, Pin1 behaves as a lamin A/C PPIase *in vitro* causing an increase in the interconversion rate of Pro23 in a Ser22-phosphorylated state of the peptide.

### The Pin1-induced isomerization directed to the pSer/Thr-Pro motif is predicted to affect the overall structure of the N-terminus of nuclear lamins

Since NMR data revealed that Pin1 activity increases the interconversion rate of Pro23, we performed molecular dynamics (MD) simulations to investigate the structural effect of Ser22 phosphorylation and Pro23 isomerization. For this purpose, three different peptides spanning residues 1–30 of lamin A/C that differ either in the phosphorylation state of Ser22 or the isomerization state of Pro23, i.e. (i) with Ser22 not phosphorylated and Pro23 in *trans* configuration (Ser22 / *trans* Pro23), (ii) Ser22 phosphorylated and Pro23 in *trans* configuration (pSer22/Pro23 *trans*), and (iii) Ser22 phosphorylated and Pro23 in *cis* configuration (pSer22/Pro23 *cis*), were simulated. A comparison of the Ser22/Pro23 *trans* and pSer22/Pro23 *trans* peptides revealed that phosphorylation itself has only a small effect and both peptides represented similar conformations over the simulation time. This is evidenced by the histogram in Fig 4C. As one minor difference, we noted a slight decrease in the helix stability of residues 23–30 upon Ser22 phosphorylation (Fig 4D, left and middle panel), which most likely results from electrostatic interactions of phosphorylated Ser22 with the adjacent arginines, thus favouring alternative backbone conformations. A comparison of the pSer22/Pro23 *trans* and pSer22/Pro23 *cis* peptides revealed that Pro23 *cis/trans* isomerization has a more pronounced effect on the overall shape of the lamin A/C N-terminus. For Pro23 in *cis*, a kink emerges in the lamin A/C structure, which causes the sampling of less extended peptide conformations (Fig 4D, middle and right panel). This is also evident from the histogram showing that the length of the most prevalent peptide conformations is reduced by more than 10% from 35 Å to 31 Å (Fig 4C). Therefore, an increased interconversion between the Pro23 *cis* and *trans* isomers might have an impact on the structure of lamin A/C, thereby also affecting its ability to form lamin multimers as a requirement to constitute a structurally interconnected nuclear lamina.

### The nuclear lamina of Pin1 knockout cells is resistant to kinase-induced disassembly

**Fig 5:**
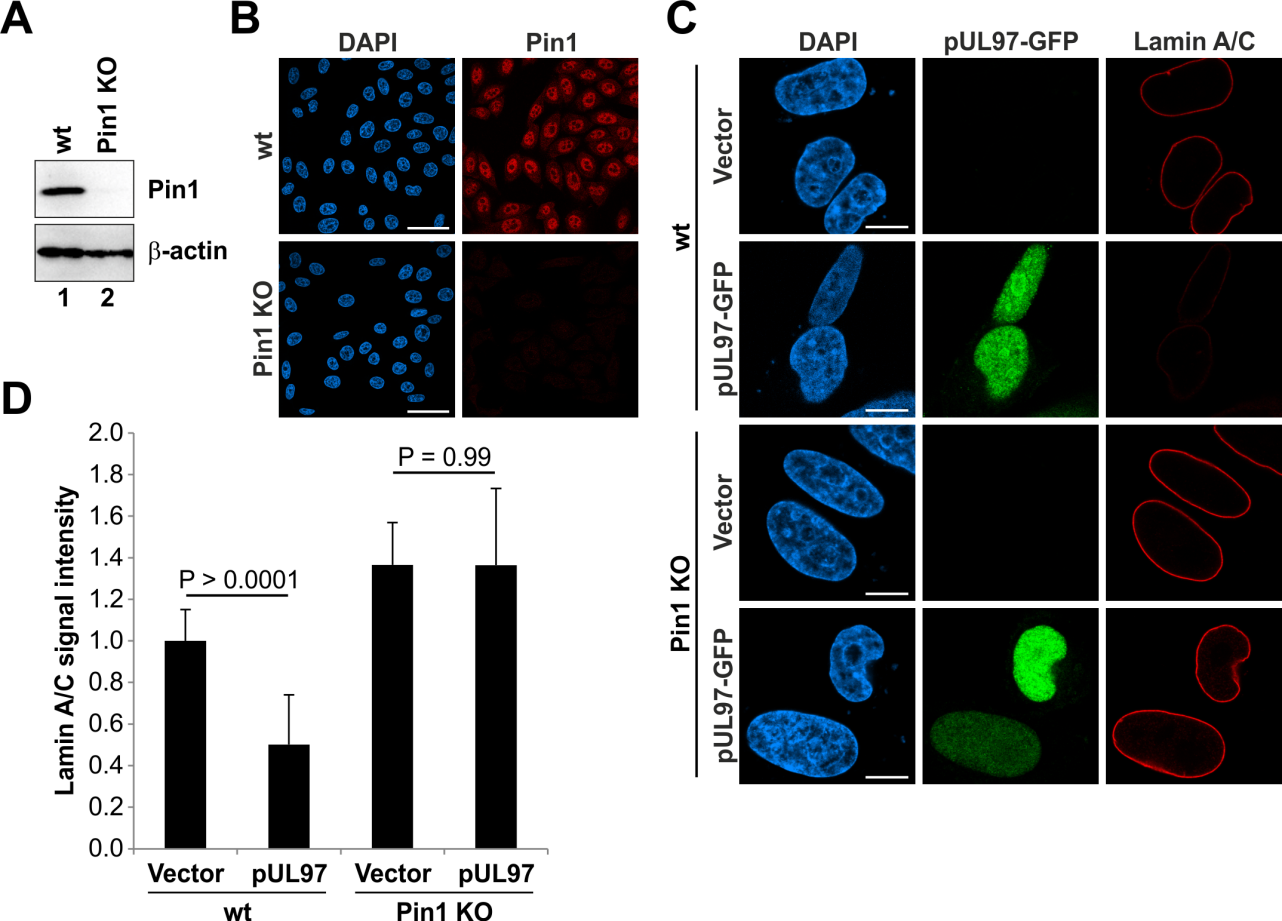
Lamin phosphorylation by transiently expressed pUL97 kinase is not sufficient for lamina disassembly in Pin1 KO cells. (A-B) Monitoring of the Pin1 KO in HeLa cells. (A) Total cell lysates of wt and Pin1 KO HeLa cells were subjected to standard Western blot analysis for detection of Pin1 (rabbit pAb-Pin1; upper panel) and loading control β-actin (lower panel). (B) Confocal microscopic images of fixed wt and Pin1 KO HeLa cells stained with rabbit pAb-Pin1 and DAPI. *Scale bars*, 50 μm. (C) Wt HeLa cells and Pin1 KO HeLa cells were transiently transfected with pcNDA3.1 (vector) or a plasmid coding for HCMV pUL97 fused to GFP. Cells were fixed at 24 h post-transfection followed by counterstaining of cell nuclei with DAPI. Samples were analysed by confocal microscopy. *Scale bars*, 10 μm. (D) Quantitation of lamin A/C signals. Signal intensities from raw images were measured along the nuclear rim (mean of ≥ 20 cells in each case, ± standard deviation; statistical significance was determined by Student’s t-test).

To substantiate the hypothesis that a Pin1-induced conformational change of lamins may result in lamina disassembly, we first analysed if the nuclear lamina is sensitive to kinase-mediated disassembly in absence of Pin1. Therefore, we generated Pin1 knockout (KO) HeLa cells using the CRISPR/Cas9 system. Efficiency of the Pin1 KO was monitored by Western blot and immunofluorescence analyses (Fig 5A and 5B). The morphology of the nuclear lamina was investigated by confocal microscopy and visualized effects produced by the expression of the HCMV kinase pUL97 in wild-type (wt) and Pin1 KO HeLa cells (Fig 5C and 5D). Previously, it has been shown that overexpressed HCMV pUL97 is generally able to induce lamina disassembly in transiently transfected cells [16,24]. In this study, only wt Hela cells showed a decrease of endogenous lamin signals upon expression of HCMV pUL97, while the nuclear lamina of Pin1 KO HeLa cells remained unaffected (Fig 5C). The endogenous lamin A/C staining of vector-transfected showed a strict rim signal in both wt and Pin1 KO cells (Fig 5C). Quantitation of signal intensities further indicated that lamin A/C levels are generally higher in Pin1 KO compared to wt HeLa cells (Fig 5D). This might suggest that the nuclear lamina is more densely packed in absence of Pin1.

### The negative charge conferred by Ser22 phosphorylation is not sufficient for lamina disassembly

**Fig 6:**
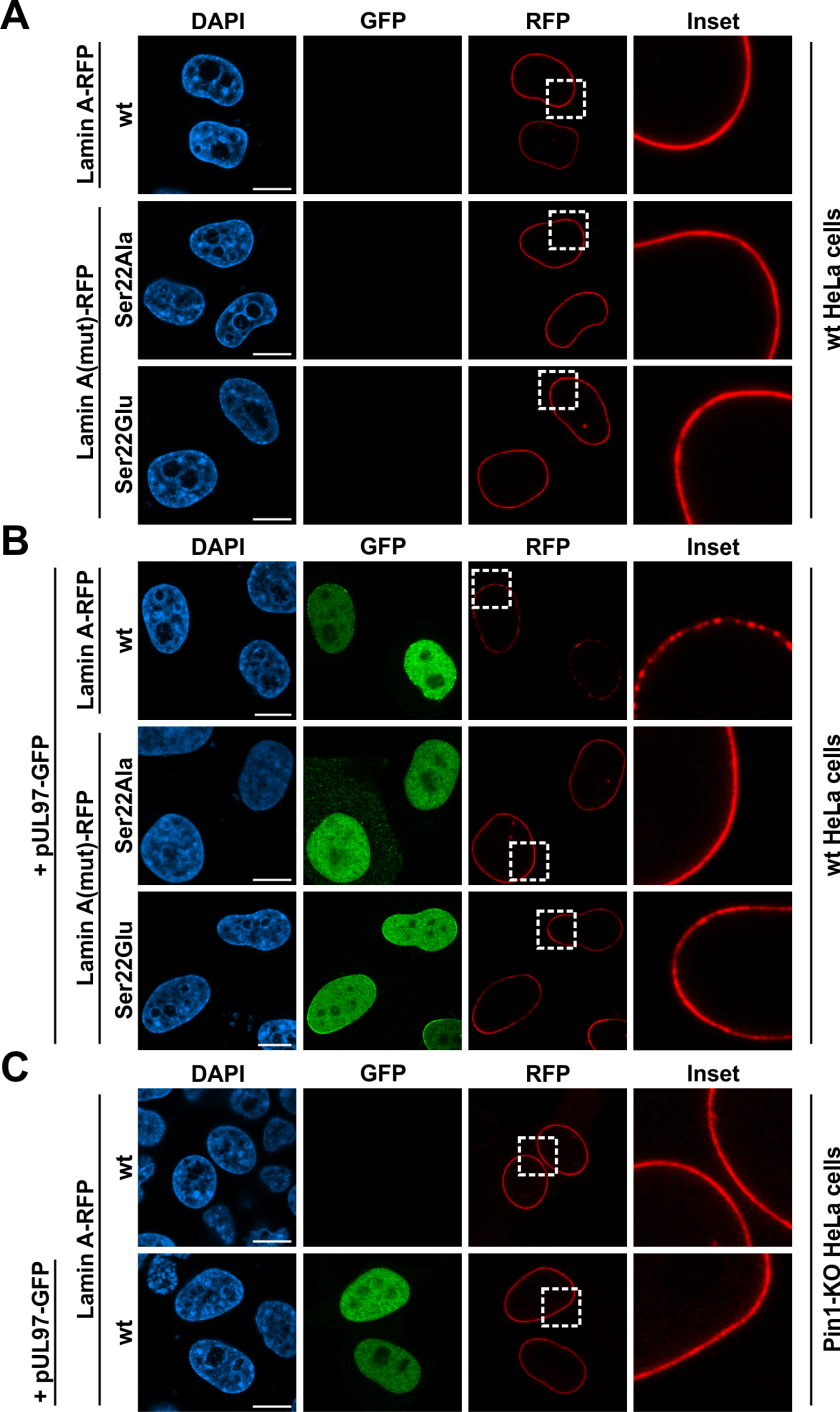
Effect of HCMV pUL97 coexpression on the localization of wild-type lamin A and phospho-mimetic and -deficient mutants. Wt HeLa cells (A-B) and Pin1 KO HeLa cells (C) were transiently transfected with plasmids coding for HCMV pUL97 fused to GFP and wt or mutant lamin A fused to RFP as indicated. Cells were fixed at 24 h post-transfection followed by counterstaining of cell nuclei with DAPI. Samples were analysed by confocal microscopy. Insets show the magnification of dashed boxes. *Scale bars*, 10 μm.

In a next step, we generated red fluorescent protein (RFP)-fused lamin A mutants which do not generate a Pin1-binding motif upon phosphorylation of Ser22. Since mutation of Pro23 is likely to affect the overall structure of the lamin N-terminus, we substituted Ser22 with glutamic acid (Glu) or alanine (Ala) to produce a phosho-mimetic (Ser22Glu) and a phospho-deficient (Ser22Ala) lamin A mutant, respectively. To test whether the negative charge conferred by Ser22 phosphorylation was sufficient to induce lamina disassembly, we analysed the localization of these lamin mutants by confocal microscopy (Fig 6). Intriguingly, the phospho-mimetic lamin A mutant Ser22Glu showed a uniform localization along the nuclear rim of transfected wt HeLa cells similar to the phosho-deficient mutant Ser22Ala or wt lamin A (Fig 6A). This indicated that the phospho-mimetic lamin A mutant cannot be recognized by Pin1 which is consistent with the observation of Moretto-Zita *et al*. [33] describing the failure of Ser-to-Glu mutants of the transcription factor Nanog to interact with Pin1. Moreover, coexpression with HCMV pUL97 induces a disruption of the uniform staining of wt lamin A illustrated by dot-like accumulations along the nuclear rim (Fig 6B). Importantly, the phospho-mimetic and -deficient lamin A mutants were both resistant to the pUL97-mediated drastic redistribution observed for wt lamin A, however, the Ser22Glu mutant showed a thinning of the lamin staining at certain areas (Fig 6B). These minor alterations in the localization of the phospho-mimetic mutant might be explained by the fact that pUL97 can phosphorylate lamin A/C at further sites besides Ser22, at least *in vitro* [19]. Confirming the effect on endogenous lamin A/C (Fig 5C and 5D), pUL97 was also not able to disrupt the rim staining of overexpressed wt lamin A in Pin1 KO HeLa cells (Fig 6C and S6 Fig). Taken together, the negative charge conferred by Ser22 phosphorylation is not sufficient for lamina disassembly, but Ser22 phosphorylation appears to be a prerequisite for the activity of Pin1.

### Pharmacological inhibition of Pin1 activity partially restores the integrity of the nuclear lamina in HCMV-infected cells

**Fig 7:**
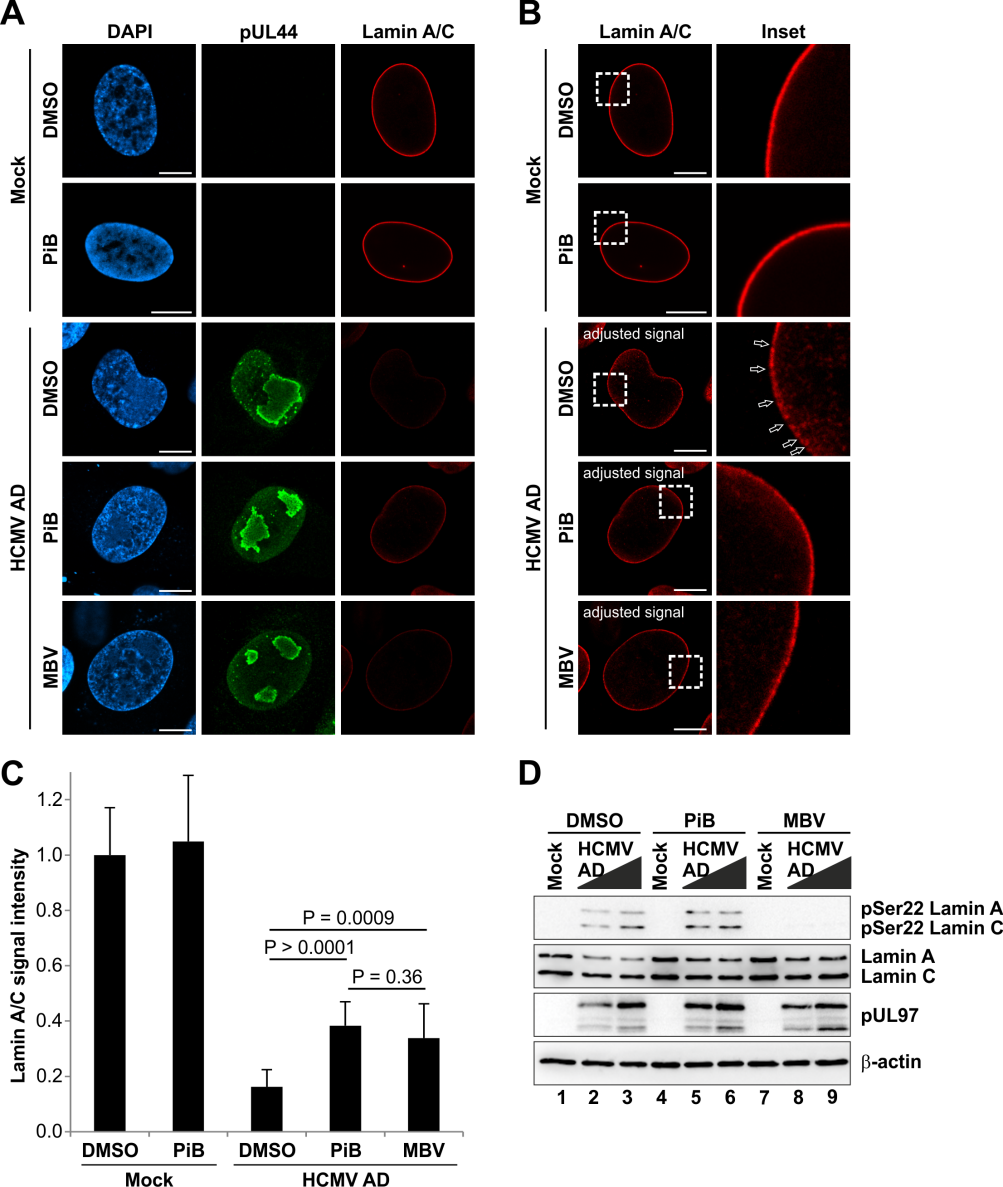
Reduced lamina disassembly upon inhibition of Pin1 activity in HCMV-infected cells. HFFs were infected with HCMV AD at a MOI of 0.01 or remained uninfected (mock). At 48 hpi, cells were treated with DMSO, 10 μM PiB or 5 μM MBV as indicated. Cells were fixed at 72 hpi followed by immunofluorescence staining using rabbit mAb-lamin A/C to visualize the nuclear lamina in red. Staining of HCMV pUL44 served as a viral marker in green. (A) Representative images of confocal planes demonstrating the effect of inhibitory compounds on the distribution of lamin A/C (raw images). (B) Lamin A/C signals of images depicted in (A) were adjusted to levels of uninfected control cells treated with DMSO for qualitative analysis of lamin A/C distribution. Insets show the magnification of dashed boxes. *Open arrows*, lamina-depleted areas; *scale bars*, 10 μm. (C) Quantitation of lamin A/C signals. Signal intensities from raw images were measured along the nuclear rim (mean of 10 cells in each case, ± standard deviation; statistical significance was determined by Student’s t-test). (D) Effect of PiB on the HCMV-induced Ser22 phosphorylation. HFFs were infected with HCMV at MOIs of 0.1 and 1.0. At 48 hpi, cells were treated with DMSO, 10 μM PiB, and 5 μM MBV. Cells were lysed at 72 hpi and lysates were subjected to standard Western blot analysis for detection of lamin A/C phosphorylated at Ser22 (upper panel), total lamin A/C (second panel), viral protein kinase pUL97 (third panel), and loading control β-actin (lower panels).

To investigate whether Pin1 is also required for lamina disassembly during herpesviral infection, we inhibited Pin1 activity in HCMV-infected cells and analysed the integrity of the nuclear lamina by confocal immunofluorescence microscopy (Fig 7). Since Pin1 might also affect different phases of herpesviral replication besides nuclear egress, the inhibitor treatment was not started immediately after virus infection as performed for the GFP-based replication assay (Fig 3C). Notably, local lamina disassembly (i.e. lamina-depleted areas) was detected in HCMV-infected cells at 72 hpi and later [16]. Thus, the Pin1 inhibitor PiB was provided at 48 hpi to ensure that Pin1 activity was inhibited before the major onset of virus-induced lamina disassembly. Addition of PiB at 48 hpi did not affect levels of viral marker proteins in Western blot analysis (S7 Fig). This suggests that early viral regulatory steps preceding nuclear egress were not or only poorly inhibited by this experimental strategy. Intriguingly, lamina disassembly, typically detectable by decreased lamin A/C signals at the nuclear rim of HCMV-infected cells, was attenuated in the presence of PiB (Fig 7A and 7C). Moreover, local depletion of the nuclear lamina was also impaired upon PiB treatment (Fig 7B). Notably, similar effects were detected by application of the HCMV pUL97 kinase inhibitor maribavir (MBV) (Fig 7A–7C). To exclude a direct effect of PiB on pUL97 activity during infection, we performed comparative Western blot analysis with lysates from HCMV-infected cells treated at 48 hpi with PiB, MBV, or the solvent control DMSO (Fig 7D). Importantly, PiB treatment neither affected Ser22 phosphorylation levels nor expression of pUL97 (Fig 7D and S7 Fig). These findings additionally confirmed that the inhibitory effect of PiB was directed to the late phase of HCMV replication, particularly to nuclear egress including the phosphorylation-dependent disassembly of the nuclear lamina. Moreover, we analysed the subcellular localization of the INM protein emerin to exclude a general effect of PiB on nuclear envelope integrity. Of note, we observed no differences in the regular rim staining of emerin at the nuclear envelope of uninfected (mock) and HCMV-infected cells treated with PiB (S8 Fig). In conclusion, Pin1 activity is essential for efficient lamina disassembly and the induction of lamina-depleted areas in HCMV-infected cells.

### Association of phosphorylated lamins with the nuclear lamina upon inhibition of Pin1 activity

**Fig 8:**
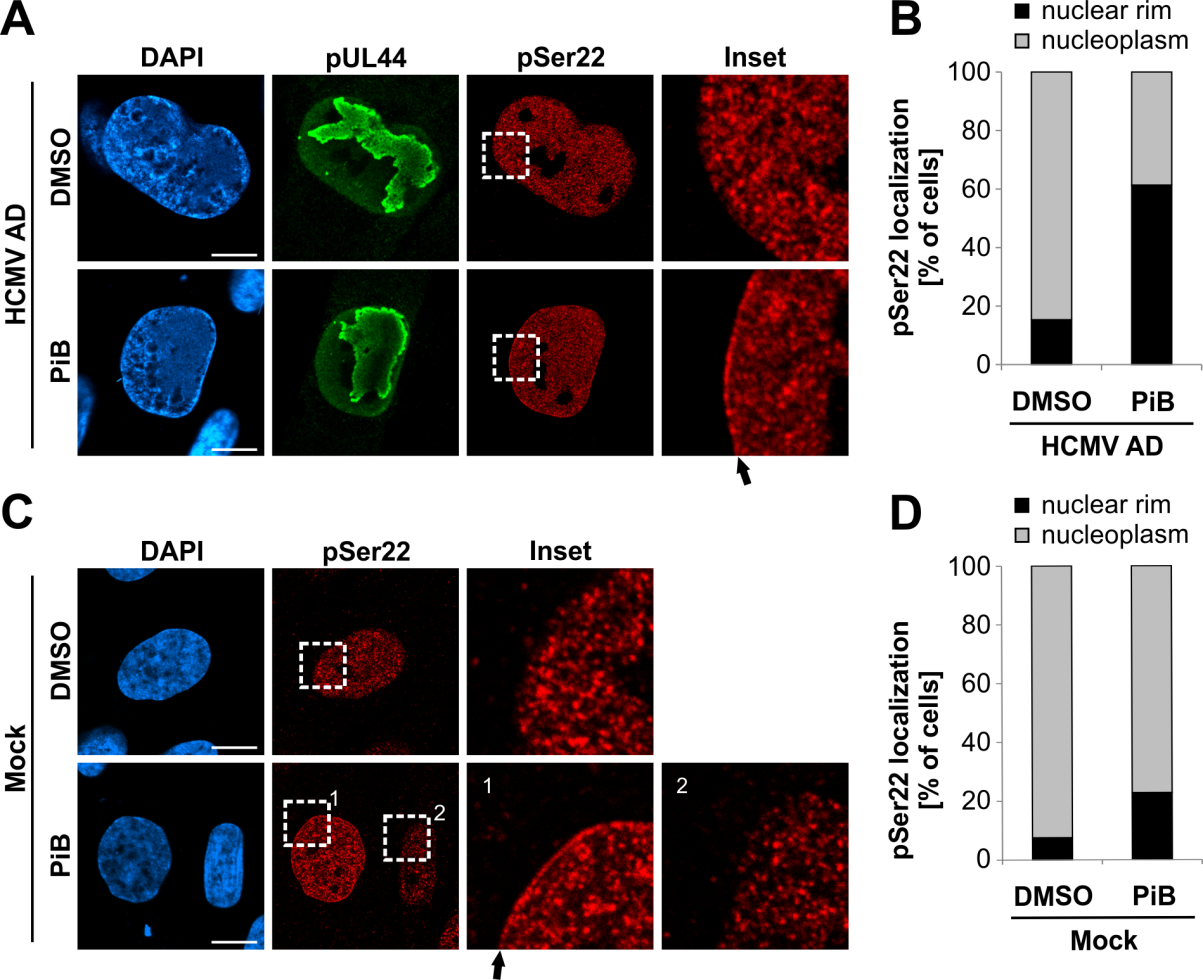
Pharmacological inhibition of Pin1 results in the accumulation of phosphorylated lamins at the nuclear rim. HFFs were infected with HCMV AD at a MOI of 0.01 (A-B) or remained uninfected (mock) (C-D). At 48 hpi, cells were treated with DMSO or 10 μM PiB as indicated. Cells were fixed at 72 hpi followed by immunofluorescence staining using a phospho-specific antibody to detect lamin A/C phosphorylated at Ser22. Staining of HCMV pUL44 served as a viral marker in green. (A,C) Representative confocal images illustrate partial relocalization of phosphorylated lamins to the nuclear envelope in cells treated with PiB. Insets show the magnification of dashed boxes. *Filled arrows*, accumulation of pSer22 lamin A/C at the nuclear envelope; *scale bars*, 10 μm. (B,D) Cells with lamina-associated pSer22 lamin A/C relative to cells with soluble pSer22 lamin A/C diffusely localized in the nucleoplasm. The percentage of cells showing these phenotypes was determined by scoring ≥ 39 cells for each setting.

Phosphorylation of lamins at ‘mitotic sites’ disassembles the nuclear lamina and causes a diffuse nuclear localization of lamin A/C (Fig 2A and S1A Fig) [1]. If a Pin1-induced conformational change is responsible for lamina disassembly, then Pin1 inhibition should reduce phosphorylated lamin A/C dissolving away from the nuclear lamina. Thus, finally, we analysed the localization of phosphorylated lamin A/C in the presence of the Pin1 inhibitor PiB (Fig 8). Using confocal immunofluorescence microscopy, we observed an accumulation of Ser22-phosphorylated lamin A/C at the nuclear rim of HCMV-infected cells treated with PiB at 72 hpi (Fig 8A). This phenotype was detected in 61.4% of infected cells upon PiB treatment, in contrast to 15.4% of dimethyl sulfoxide (DMSO)-treated control cells (Fig 8B). Here again, PiB treatment was started at 48 hpi to reduce effects on other phases of viral replication besides nuclear egress. Interestingly, we also detected increased accumulation of Ser22-phosphorylated lamin A/C in a subset of uninfected cells treated with PiB for 24 h (Fig 8C). In this experiment, we evaluated uninfected interphase cells with detectable levels of Ser22 phosphorylation indicating imminent cell division [34]. In this setting, 10.4% of cells showed pSer22 staining exclusively at intranuclear locations. Of these cells, 22.9% of PiB-treated cells in contrast to 7.6% of DMSO-treated control cells showed Ser22 staining at the nuclear rim (Fig 8D). Taken together, inhibition of Pin1 during HCMV replication, as well as in uninfected cells, prevents Ser22-phosphorylated lamin A/C from becoming dispersed throughout the nucleoplasm suggesting an association with the nuclear lamina despite its phosphorylated state.

## Discussion

Although site-specific lamin phosphorylation has been known for a long time to cause disassembly of the nuclear lamina, the underlying mechanism is still not completely understood. It has been proposed that phosphorylation interferes with electrostatic interactions between lamin dimers, thereby promoting lamina disassembly. This scenario was challenged by the identification of a phosphorylation-dependent binding motif for the PPIase Pin1 in lamins and that Pin1 might induce a conformational change to facilitate lamina disassembly [16,35]. In this study, we used herpesvirus-mediated lamin phosphorylation during nuclear egress as a tool to analyse the role of Pin1 in lamina disassembly. Consistent phosphorylation of the ‘mitotic site’ Ser22 in infected cells suggested a common mechanism of lamina disassembly which is conserved among herpesviruses and might involve Pin1. Notably, we demonstrated isomerization of a Ser22-phosphorylated lamin A/C peptide by Pin1 *in vitro* using NMR spectroscopy. Bioinformatics analysis further suggested that Pin1-mediated *cis/trans* isomerization of Pro23 induces a kink in the lamin N-terminus. Cell culture experiments finally provided evidence that the presence of Pin1 and its PPIase activity are essential for the efficient disassembly of the nuclear lamina. Thus, we propose a model in which Pin1 facilitates depolymerization of lamin filaments by inducing a conformational change in nuclear lamins.

As a main finding of the present study, Ser22 and Ser392 phosphorylation of lamin A/C was consistently detected for several herpesviruses, although quantitative differences were identified. While Ser22 phosphorylation has been constantly found for various herpesviruses, increase of Ser392 phosphorylation, compared to uninfected cells, has been observed mostly for the alphaherpesvirus HSV-1. Earlier reports suggested that cellular protein kinases, such as PKC isoforms, are specifically recruited during herpesviral infection to phosphorylate nuclear lamins [36–38]. In more recent reports, it was shown that virus-encoded protein kinases, such as HCMV pUL97, HSV-1 pUS3, and others, are involved in lamin phosphorylation [16,19,39–41]. Accordingly, we found in the present study that high levels of Ser22 phosphorylation were missing in cells infected with a UL97-deficient HCMV. In contrast, infection with a US3-deficient HSV-1 resulted in a limited but still detectable increase in Ser22 phosphorylation compared to uninfected cells. Notably, alphaherpesviruses encode two protein kinases such as pUS3 and pUL13, belonging to the HvUS and HvUL group, respectively [14], while beta- and gammaherpesviruses exclusively encode HvUL group homologues such as HCMV pUL97 (also termed as conserved herpesvirus protein kinases, CHPKs) [42]. As reported by Kuny *et al*. [24], transient overexpression of HvUL group homologues of human beta- and gammaherpesviruses, but not those of alphaherpesviruses, resulted in a Ser22-specific phosphorylation of lamins leading to nuclear lamina disassembly in plasmid-transfected cells. Thus, the consistent phosphorylation of lamin A/C at Ser22 in herpesvirus-infected cells demonstrated in the present study indicates that different lamin-directed viral and probably also cellular protein kinases may act in concert to modify the nuclear lamina.

The lamin phosphorylating viral and cellular protein kinases are recruited to the nuclear lamina by viral egress core proteins to form a multimeric nuclear egress protein complex (NEC) [8,14,15]. The core of this NEC is formed by a heterodimer of the two conserved herpesviral nuclear egress proteins, designated as pUL50 and pUL53 in the HCMV nomenclature [43]. The core NEC serves as a scaffold for the association of further viral and cellular NEC components [44]. Proteomic analyses recently provided candidate lists of proteins contained within the NECs of cells infected with human or murine cytomegaloviruses [45,46]. Of note, it was demonstrated that the NEC has additional activities beyond the regulation of nuclear lamina disassembly. The core NEC is sufficient for membrane deformation and scission which is necessary for budding of viral capsids at the INM, a process which requires previous lamina disassembly [47–49].

CDK1-mediated phosphorylation of Ser22 and Ser392 is associated with lamina disassembly during mitosis. In particular, based on the crystal structure of a lamin A fragment, it has been suggested that phosphorylation of Ser22 and, possibly, Ser392 interferes with electrostatic interactions between the head and tail domains with the N- and C-terminal regions of the rod domain, respectively, and thereby initiates lamin disassembly down to the dimeric level [50]. The presented data of this study now suggests that phosphorylation alone is not sufficient for lamina disassembly *in vivo*. In fact, phosphorylation at Ser22 might be required for a Pin1-induced conformational change of lamin A/C which finally triggers depolymerization of lamin filaments. Confirming this hypothesis, we demonstrated that the phospho-mimetic Ser22Glu lamin A mutant was still able to form a regular lamina structure and that a Ser22-phosphorylating kinase did not induce lamina disassembly in absence of Pin1. In addition, we showed that pharmacological inhibition of Pin1 activity interferes with efficient lamina disassembly during nuclear egress of herpesviral capsids. Alternatively, the effect of Pin1 inhibition or Pin1 knockout might also be explained by the possibility that the Pin1-catalysed conformational change keeps lamins in a phosphorylated state and thereby dissolved from the nuclear lamina. In this scenario, the compact conformation of lamin A/C with a *cis* Pro23 might impede lamin dephosphorylation by phosphatases which would be required for reassembly of the nuclear lamina [1]. Importantly, activity of Pro-directed phosphatases is configuration specific and substrate residues can only be dephosphorylated in the *trans* isomers [17]. However, our observation that Pin1 inhibition results in the association of Ser22-phosphorylated lamin A/C with the nuclear lamina favours the setting of a conformational change-driven depolymerization of lamin filaments or even a combination of both scenarios.

During cell cycle progression, Pin1 activity is regulated by post-translational modification [17]. In particular, Pin1 is inactivated by phosphorylation at Ser16 during G2/M transition. After entry into mitosis, Pin1 activity is restored by the dephosphorylation of Ser16 and the activating phosphorylation at Ser65 [51]. Thus, Pin1 is in an active state during mitosis to facilitate lamina disassembly. At the end of mitosis, the nuclear lamina reassembles along with the reformation of the nuclear envelope. Dephosphorylation of lamins is required for lamina reassembly [52]. Inactivation of Pin1 might be, therefore, required to shift the balance to the *trans* isomer to allow lamin dephosphorylation by phosphatases [17]. In herpesvirus-infected cells, we detected increased Pin1 expression. Analysis of post-translational modifications in future studies might reveal similar mechanisms of Pin1 regulation as seen during mitosis.

In addition to the targeting of Pin1 to lamins during nuclear egress, Pin1 might also have importance for further regulatory steps in herpesviral replication. It was demonstrated for the EBV DNA polymerase catalytic subunit BALF5 that it interacts with Pin1 in GST pulldown and immunoprecipitation assays. Since the DNA replication defect of a recombinant BALF5-deficient virus could be efficiently complemented only by exogenous supply of wild-type BALF5, but not by a binding-deficient BALF5 mutant, a regulatory role for Pin1 in herpesviral DNA replication was postulated [53].

**Fig 9:**
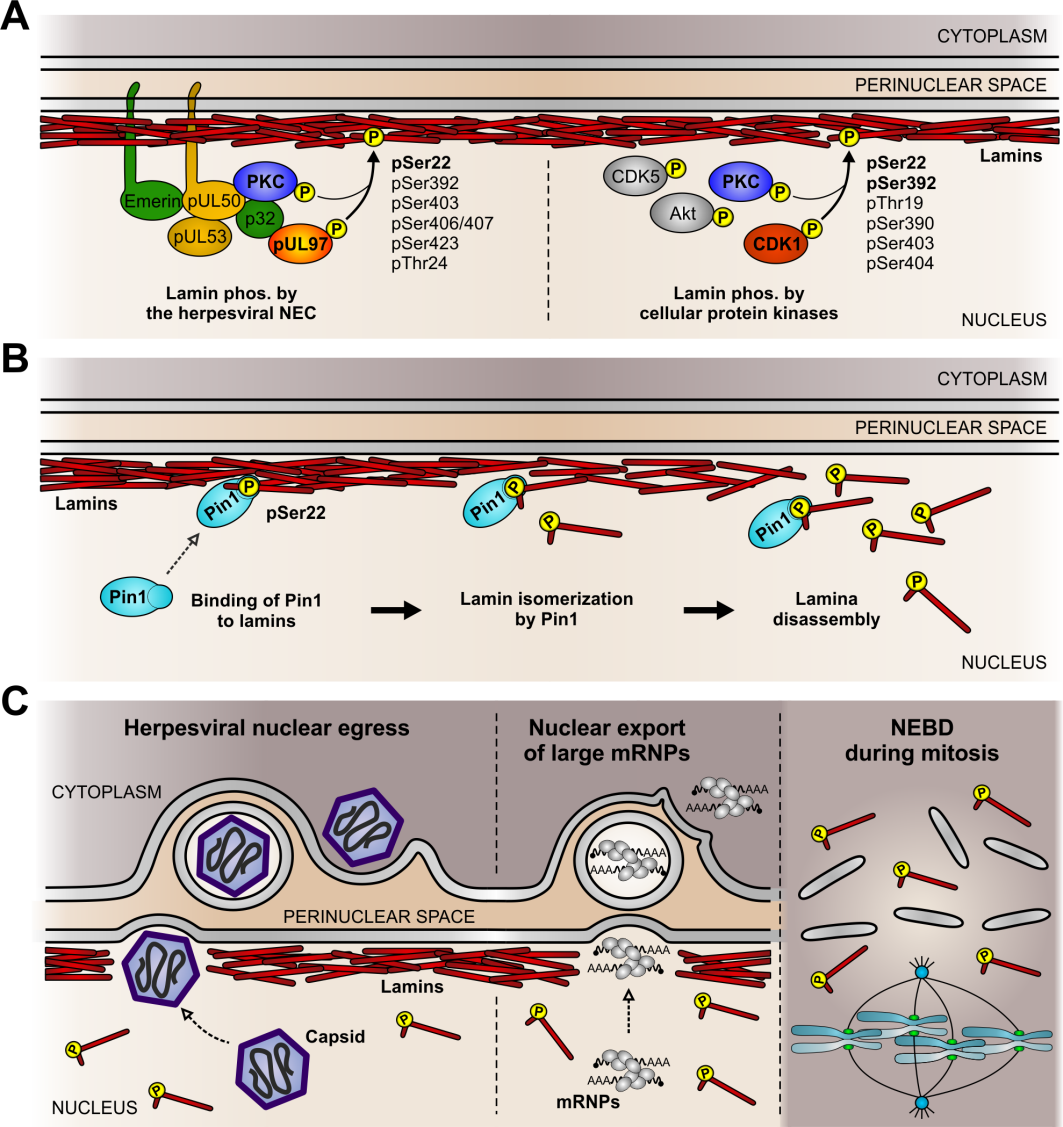
Putative conserved mechanism of Pin1-induced nuclear lamina disassembly. (A) Site-specific lamin phosphorylation by herpesvirus-encoded or host cell protein kinases. *NEC*, nuclear egress complex; *PKC*, protein kinase C; *CDK*, cyclin-dependent kinase. (B) Lamina disassembly as a direct consequence of a Pin1-mediated conformational change of lamins. (C) Cellular processes which require disassembly of the nuclear lamina. *mRNP*, messenger ribonucleoprotein complexes; *NEBD*, nuclear envelope breakdown. Diagram not to scale.

In conclusion, our findings provide evidence for a conserved phosphorylation-triggered mechanism of lamina distortion during herpesviral nuclear egress. The presented data provide evidence for a specific functional role for Pin1 in herpesvirus-induced lamina disassembly. Our findings also suggest a functional model of similar events that mediate lamina disassembly during mitosis or the non-canonical nuclear export of large mRNP complexes in uninfected cells (Fig 9). Specifically, site-specific lamin phosphorylation by herpesvirus-encoded or host cell protein kinases initiates lamina disassembly (Fig 9A). In HCMV-infected cells, cellular (e.g. PKC) and viral protein kinases (e.g. pUL97) are recruited to the nuclear lamina by the viral NEC (Fig 9A, left). In uninfected cells, lamins can be phosphorylated by various endogenous protein kinases (Fig 9A, right). In both cases, Ser22-specific lamin phosphorylation generates a Pin1-binding motif. After binding, Pin1-mediated *cis/trans* isomerization of Pro23 then induces a conformational change in the lamin N-terminus resulting in local lamina disassembly (Fig 9B). In herpesvirus-infected cells, the produced disassembly of the nuclear lamina enables budding of viral capsids at the INM for nuclear egress (Fig 9C, left). Intriguingly, the lamina disassembly during the herpesviral nuclear egress pathway appears to be similar to the lamina disassembly required for nuclear envelope budding of large mRNP complexes (Fig 9C, middle) and for nuclear envelope breakdown during mitosis (Fig 9C, right). These novel insights into the concerted action of protein kinases and Pin1 improve our understanding of various effector functions controlling nuclear herpesvirus-host interaction.

## Methods

### Cells

Primary human foreskin fibroblasts (HFFs; already-existing collection of Manfred Marschall’s laboratory) were cultivated in minimal essential medium (Thermo Fisher Scientific) containing 7.5% fetal calf serum, 350 μg glutamine per ml, and 100 μg gentamicin per ml. Wild-type (wt) HeLa cells (already-existing collection of Manfred Marschall’s laboratory) were cultivated in Dulbecco's modified Eagle medium (DMEM; Thermo Fisher Scientific) containing 10% fetal calf serum, 350 μg glutamine per ml, and 100 μg gentamicin per ml. Pin1 knockout (KO) HeLa cells were generated by cotransfection of plasmids encoding the Cas9 nuclease and a pool of three Pin1-specific 20 nt guide RNAs together with the corresponding homology-directed repair plasmids (Santa Cruz Biotechnology) according to the manufacturer’s protocol. Pin1 KO HeLa cells were selected with DMEM containing 5 μg per ml puromycin, 10% fetal calf serum, 350 μg glutamine per ml, and 100 μg gentamicin per ml. Efficiency of the Pin1 KO was controlled by Western blot staining and immunofluorescence analysis.

### Viruses

For infection experiments, HFFs (passage no. 10–15) were grown in 12-well plates at a density of 2.25x10^5^ cells per well. HFFs were infected at MOIs between 0.01 and 3.0 with virus-containing supernatants of HFFs infected with either HCMV laboratory strain AD169 (HCMV AD; already-existing collection of Manfred Marschall’s laboratory), AD169-derived UL97-deficient HCMV (HCMV ΔUL97; already-existing collection of Manfred Marschall’s laboratory), or recombinant HCMV strain TB40 (HCMV TB; kindly provided by Christian Sinzger, Ulm, Germany) expressing the capsid-associated protein pUL32 fused with GFP as described previously [16]. Similarly, HFFs were infected under identical conditions with herpes simplex virus type 1 (HSV-1; kindly provided by Peter O’Hare, London, UK), US3-deficient HSV-1 (HSV-1 ΔUS3; kindly provided by Beate Sodeik; Hannover, Germany), rhesus macaque CMV (RhCMV; kindly provided by Amitinder Kaur, Boston, MA, USA), or murine herpesvirus 68 (MHV-68; kindly provided by Heiko Adler, München, Germany). For the highly cell-associated varicella zoster virus (VZV) and human herpesvirus 6A (HHV-6A) [54,55], HFFs were infected by cocultivation with serial dilutions of VZV-infected HFFs (kindly provided by Klaus Korn, Erlangen, Germany) or HHV-6A-infected J-Jhan cells (immortalized human T lymphocytes; already-existing collection of the laboratories of Yasuko Mori and Benedikt Kaufer) for five or three days, respectively. The infected cells were harvested for subsequent analysis at 20–24 hpi (HSV-1, HSV-1 ΔUS3), 72 hpi (HCMV AD, HCMV TB, HHV-6A, RhCMV, MHV-68), or 120 hpi (VZV), unless otherwise indicated. Characteristic features of the viruses used are depicted in Table 1.

### Expression plasmids and transient transfection

An expression plasmid, encoding a fusion of lamin A and the red fluorescent protein (RFP), was generated by subcloning of the lamin A open reading frame from the template pS65T-lamin A (kindly provided by Dr. J. Broers, Cardiovascular Research Institute, University of Maastricht, Maastricht, The Netherlands) into the destination vector pDsRed2-C1 (Clontech Laboratories) by cleavage with EcoRI/BamHI. In addition, expression constructs coding for mutant lamin A carrying a single amino acid exchange of serine 22 to alanine (Ser22Ala) or glutamic acid (Ser22Glu) were generated using the GeneArt Site-Directed Mutagenesis System (Thermo Fisher Scientific) according to the manufacturer’s protocol. Site-directed mutagenesis PCR was performed with pDsRed2-C1 lamin A as a template and oligonucleotide primers with nucleotides differing from the wild-type sequence; sequences of oligonucleotides purchased from Biomers (Ulm, Germany) are given in supplemental S1 Table. An expression plasmids coding for FLAG-tagged HCMV kinase pUL97 was described previously [56]. Transfection of these expression plasmids into wt HeLa and Pin1 KO HeLa cells was performed using Lipofectamine 2000 (Thermo Fisher Scientific) according to the manufacturer’s protocol.

### Antibodies

Rabbit monoclonal antibody (mAb) anti-Lamin A (EPR4100; Abcam plc) was used to detect total levels of lamin A/C. Rabbit polyclonal antibodies pAb-Lamin A/C Ser22 (Cell Signaling Technology) and pAb-Lamin A/C (S392; Abcam plc) were used to detect lamins A/C when phosphorylated at Ser22 or Ser392, respectively. Additional antibodies against cellular proteins were rabbit mAb-Pin1 (EP1479Y; Novus Biologicals), rabbit pAb-Pin1 (H-123; Santa Cruz Biotechnology), mouse mAb-emerin (H-12; Santa Cruz Biotechnology), and mouse mAb-β-actin (AC-15; Sigma-Aldrich). Further antibodies against viral proteins were (a) HSV-1, mouse mAb-ICP0 (11060), mAb-UL42 (13C9), and mAb-ICP5 (3B6) (Santa Cruz Biotechnology); (b) VZV, mouse mAb-orf3 (3.01), mAb-orf24 (24.08), and mAb-orf27 (27.1) (kindly provided by Stipan Jonjic and Tihana Lenac Roviš, Rijeka, Croatia) [57]; (c) HCMV, mouse mAb-IE1, mAb-MCP and mAb-pp28 (kindly provided by William Britt, Birmingham, AL, USA), mouse mAb-UL44 (kindly provided by Bodo Plachter, Mainz, Germany), rabbit pAb-UL97 (Ulm; kindly provided by Detlef Michel, Ulm, Germany) [58], and mAb-UL50 (1A11; kindly provided by Stipan Jonjic and Tihana Lenac Roviš, Rijeka, Croatia) [45]; (d) HHV-6A, mouse mAb-p41/38 (C-5; kindly provided by the HHV-6 Foundation, Santa Barbara, CA, USA); (e) RhCMV, mouse mAb-gB (27–287) and rabbit pAb-RhIE1 (kindly provided by Michael Mach, Erlangen, Germany); and (f) MHV-68, polyspecific antisera from MHV-86-infected mice (kindly provided by Heiko Adler, München, Germany). In addition, mouse mAb-GFP (7.1 and 13.1; Roche Life Science) was used to detect GFP expressed during infection with recombinant viruses HSV-1, HCVM TB, HCMV ΔUL97, HHV-6A and MHV-68. Secondary antibodies were Alexa Fluor 488-/555-/647-conjugated secondary antibodies for indirect immunofluorescence (Molecular Probes) and horseradish peroxidase-conjugated anti-mouse/-rabbit secondary antibodies for Western blot analyses (Dianova).

### Pin1 inhibitors and reference compounds

The drugs used in the present study were obtained from various sources: Pin1 inhibitor B (PiB; Sigma-Aldrich), juglone (Merck Millipore), ganciclovir (GCV; Sigma-Aldrich), staurosporine (Calbiochem), and maribavir (MBV; Shanghai PI Chemicals Ltd). Due to high target specificity and low cytotoxicity [59], PiB was used to block Pin1 activity in cell culture experiments. The potent but rather toxic Pin1 inhibitor juglone [60] was used only to block Pin1 activity *in vitro* in NMR experiments.

### Virus replication and cytotoxicity assays

A GFP-based HCMV replication assay was performed as previously described [61]. In brief, HFFs were cultivated in 12-well plates (2.25 x 10^5^ cells/well) and infected with a recombinant HCMV expressing GFP (MOI of 0.2, i.e. ~25% GFP-positive cells at 7 days post-infection). Treatment with the Pin1 inhibitor PiB, the HCMV reference compound ganciclovir (GCV), or the solvent control DMSO was started immediately after virus infection. At 7 days post-infection, potential cytotoxic effects produced by the applied substances were excluded by microscopic evaluation of cell morphology. Subsequently, the cells were lysed, and the lysates were subjected to automated GFP quantitation using a Victor 1420 multilabel counter (Perkin-Elmer).

Potential cytotoxic effects produced by PiB were further evaluated by a trypan blue exclusion assay. Therefore, HFFs were seeded in 12-well plates and incubated with increasing concentrations of the Pin1 inhibitor PiB (range 10 to 60 μM) and the reference compound staurosporine (STP; 10 μM) for 24 h. Cell staining was achieved with 0.1% trypan blue for 10 min at room temperature before percentages of viable and dead cells were determined by microscopic counting (n = 3).

### Indirect immunofluorescence analysis and measurement of signal intensities

Indirect immunofluorescence staining of HFFs for confocal microscopy was performed as previously described [38]. Images were acquired using a TCS SP5 confocal laser-scanning microscope (Leica Microsystems). Cross-talk of fluorophores in multilabelling experiments was avoided by sequential scanning of separate channels. Images of a confocal plane were recorded with a pinhole of 1 airy unit and a line average of 4.

For analysing the effects of inhibitory compounds on the nuclear lamina, signal intensities of lamin A/C were quantified along the nuclear rim by the line profile tool of LAS AF software (Leica microsystems). For quantitation of lamin phosphorylation at Ser22 and Ser392, z-series were recorded along ~19 μm (z-axis) in 30 z-slices with a pinhole of 2 airy units and a line average of 2. Signal intensities were determined semi-automatically from maximum projections of z-series by Fiji/Image J [62]. Cell nuclei were segmented by thresholding the DAPI channel followed by measuring the mean signal intensity of lamin A/C phosphorylation at Ser22 and Ser392 per nucleus. Staining of viral proteins served as a marker for virus-positive cells within the population. Lamin phosphorylation signal intensities were compared between uninfected and infected cells. The distribution of data sets of individual experiments was visualized as box plots generated with BoxPlotR [63].

### Western blot analysis

Protein lysates were prepared by resuspension of cells in a sodium dodecyl sulfate (SDS)-containing buffer and subsequent thermal denaturation at 95°C for 10 min. Protein lysates were separated on SDS-containing 8–20% polyacrylamide gels followed by transfer to nitrocellulose membranes. Immunostaining was performed by the use of monoclonal or polyclonal primary antibodies and horseradish peroxidase-conjugated secondary antibodies. Protein detection was achieved by chemiluminescence using a FUJIFILM Luminescent Image Analyser LAS-1000 (FUJIFILM Europe GmbH).

### Nuclear magnetic resonance (NMR) spectroscopy

2D ^1^H total correlation spectroscopy (TOCSY) and nuclear Overhauser enhancement spectroscopy (NOESY) NMR experiments were performed at 600.13 MHz on a Bruker Avance 600 MHz instrument equipped with an UltraShield Plus magnet and a triple resonance cryoprobe with gradient unit. Individual samples were dissolved in 600 μl 50 mM aqueous phosphate buffer pH 7.0 containing 10% D_2_O (v/v), at concentrations between 1–2 mM. The 2D NMR experiments were performed at 300 K without spinning, with mixing times of 110 ms for the TOCSY experiments and 250 ms for the NOESY experiments. Efficient suppression of the water signal was achieved with application of excitation sculpting in the 1D ^1^H and the 2D ^1^H TOCSY and NOESY NMR experiments [64]. ^1^H signal assignments of the NMR spectra were achieved by identification of the individual spin systems in the 2D ^1^H TOCSY spectra, combined with observations of sequence-specific, short-distance cross-peaks (Hα-HN i, i+1) in the 2D ^1^H-^1^H NOESY spectra [65]. Readily recognizable spin systems were used as starting points for correlation of the individual spin systems observed in the TOCSY and NOESY spectra with individual residues in the peptide sequences. Data acquisition, processing, and spectral analysis were performed with Bruker Topspin 1.3 software. Interaction of Pin1 with lamin A/C peptides: After acquisition of 1D ^1^H and 2D ^1^H TOCSY and NOESY NMR spectra of pure lamin A/C peptides, 100 μl buffer solution containing catalytic amounts of Pin1 was added to the individual peptide solutions, followed by acquisition of identical series of NMR spectra (1D ^1^H and 2D ^1^H TOCSY and NOESY) to those of the pure peptides. Exchange peaks occurring in the spectra after addition of Pin1 were identified by superimposition of analogous NOESY spectra prior to and after addition of Pin1 using Bruker Topspin 1.3 software. Inhibition of prolyl *cis/trans* isomerase interaction of Pin1 with phosphorylated lamin A/C peptide by addition of juglone: The catalytic prolyl *cis/trans* isomerase interaction of Pin1 with phosphorylated lamin A/C peptide was inhibited by addition of excessive amounts of the Pin1 inhibitor juglone dissolved in 5 μl deuterated dimethylsulfoxide (DMSO-D6). We have previously shown that the presence of 1% DMSO does not inhibit catalytic prolyl *cis/trans* isomerase interaction [28]. The disappearance of NMR exchange peaks, originating from the catalytic prolyl *cis/trans* isomerase interaction of Pin1 with the phosphorylated lamin A/C peptide, after addition of juglone was revealed by superimposition of analogous NOESY spectra prior to and after addition of the inhibitor, using Bruker Topspin 1.3 software. Peptide synthesis was performed by Metabion International AG (Germany). Recombinant human Pin1 was purchased from Biotrend Chemikalien GmbH (Germany).

### Molecular dynamics simulation

The computational investigation of linear peptides with the sequence METPSQRRATRSGAQASSTPLSPTRITRLQ followed an approach similar to earlier work [66]. Three systems were set up in an initial extended conformation, differing in the phosphorylation state of Ser22 and the configuration of Pro23: (1) Ser22 not phosphorylated, Pro23 in *trans* configuration (Ser22/Pro23 *trans*); (2) Ser22 phosphorylated, Pro23 in *trans* configuration (Ser22 phos/Pro23 *trans*); and (3) Ser22 phosphorylated, Pro23 in *cis* configuration (Ser22 phos/Pro23 *cis*). All systems were subjected to 1000 steps of energy minimization in order to remove any steric clashes and to relax the peptide. The systems were then brought to the target simulation temperature of 310 K in 0.2 ns and subsequently simulated in an NPT ensemble for 200 ns with a time step of 2 fs. During the production phase, coordinate snapshots were saved every 20 ps. The parm99SB force field [67,68] with additional parameters for phospho-serine [69] was applied and a Generalized Born implicit solvent model [70] with Bondi atomic radii was used to account for the correct environmental properties. All Coulomb and van der Waals interactions were taken into account; no cut-off was applied. Structure representatives for each system were obtained via hierarchical clustering [71]. All simulations and analyses were performed with programs from the Amber14 suite [72]; structural representations were generated with VMD [73].

## Supporting Information

S1 Fig Ser392-specific phosphorylation of lamin A/C in herpesvirus-infected primary fibroblasts analysed by confocal imaging. (A) HFFs were infected with different herpesviruses or remained uninfected (mock) as indicated. Cells were fixed at 24 hpi (HSV-1 and HSV-1 ΔUS3) or 72 hpi (VZV, HCMV AD, HCMV TB, HCMV ΔUL97, HHV-6A, and RhCMV) followed by immunofluorescence analysis using phospho-specific antibodies to detect lamin A/C phosphorylated at Ser392 in red. Staining of viral proteins or the green fluorescent protein (GFP) served as viral markers in green. Cell nuclei were counterstained with DAPI (4’,6-diamidino-2-phenylindole). Samples were analysed by confocal microscopy and a representative image of the focal plane is depicted for each setting. *Filled arrows*, nuclei of virus-positive cells; *open arrows*, nuclei of virus-positive cells showing increased Ser392 phosphorylation compared to virus-negative cells; *scale bars*, 30 μm. (B) Median intracellular intensities of lamin A/C phosphorylation. pSer392 signals were determined for infected (white boxes) and surrounding uninfected cells (grey-shaded boxes) as maximum projections of confocal z-series. One representative experiment out of three is depicted for each virus presenting the values of site-specific phosphorylation as box plots. Note, Table 2 contains the mean values ± standard deviation of three independent experiments. Centre lines show the medians with box limits indicating the 25th and 75th percentiles as determined by R software. Whiskers extend 1.5 times the interquartile range from the 25th and 75th percentiles, outliers are represented by circles, and the number of evaluated cells is depicted above each box in brackets. Statistical significance was determined by Student’s t-test (*, P < 0.05; **, P < 0.01; ***, P < 0.001; *n*.*s*., not significant, P ≥ 0.05).
(TIF)

S2 Fig Ser22-specific phosphorylation of lamin A/C during different phases of HCMV replication. HFFs were infected with HCMV AD at a MOI of 1.0 or remained uninfected (mock). Cells were lysed at 24 hpi, 48 hpi, and 72 hpi. Total lysates were subjected to standard Western blot analysis for detection of lamin A/C phosphorylated at Ser22 (pSer22; upper panel), total lamin A/C (second panel), viral protein kinase pUL97 (third panel), viral immediate early protein 1 (IE1; fourth panel), viral early protein pUL44 (fifth panel), the viral late major capsid protein (MCP; sixth panel), and the loading control β-actin (lower panel).
(TIF)

S3 Fig Cell growth is not affected by PiB treatment. Primary human foreskin fibroblasts (HFFs) were treated with 10 μM PiB or dimethyl sulfoxide (DMSO) as solvent control. At 7 d post-treatment, cell proliferation was evaluated by microscopic counting of total cell numbers (n = 4). P values were determined by Student’s t-test.
(TIF)

S4 Fig Cytotoxic effects produced by the Pin1 inhibitor PiB analysed by trypan blue exclusion assay. Primary human foreskin fibroblasts (HFFs) were treated with increasing amounts of PiB, dimethyl sulfoxide (DMSO) as solvent control, or staurosporine (STP) in a toxic concentration of 10 μM. At 24 h post-treatment, the cells were stained with trypan blue followed by microscopic counting of living and dead cells (n = 3). (A) Mean percentage of living (grey bars) and dead (black bars) cells. (B) Mean total cell numbers comprising dead and living cells.
(TIF)

S5 Fig NMR spectroscopy of lamin A/C peptides in the presence or absence of Pin1 and the Pin1 inhibitor juglone. Superimposed expanded HN-HN regions of the 2D ^1^H-^1^H NOESY spectra are depicted for phosphorylated and unphosphorylated versions of a lamin A/C peptide comprising amino acids 11–40. (A) Phosphorylated peptide after addition of Pin1 (blue signals) and after additional treatment with the Pin1 inhibitor juglone (red signals); note that prolyl *cis/trans* related exchange peaks detected after addition of Pin1 disappear after treatment with juglone. (B) Phosphorylated peptide prior to (blue signals) and after addition of both Pin1 and the Pin1 inhibitor juglone (red signals); note that the spectrum recorded after addition of both Pin1 and the Pin1 inhibitor juglone resembles the spectrum of the pure peptide.
(TIF)

S6 Fig Coexpression of the HCMV kinase pUL97 does not affect the localization of wild-type and mutant lamin A in Pin1 knockout cells. Pin1 knockout (KO) HeLa cells were transiently cotransfected with plasmids coding for HCMV pUL97 fused to the green fluorescent protein (GFP) and wild-type (wt) or mutant lamin A fused to the red fluorescent protein (RFP) as indicated. Cells were fixed at 24 h post-transfection followed by counterstaining of cell nuclei with DAPI (4’,6-diamidino-2-phenylindole). Samples were analysed by confocal microscopy. Insets show the magnification of dashed boxes. *Scale bars*, 10 μm.
(TIF)

S7 Fig Effect of PiB treatment started at 48 hpi on the expression of viral proteins. Primary human foreskin fibroblasts (HFFs) were infected with the human cytomegalovirus (HCMV) strain AD169-GFP and treated with the Pin1 inhibitor PiB added at 48 hpi at indicated concentrations of 5 or 20 μM. Western blot analysis was performed with total lysates taken at 72 hpi. Note that PiB addition at 48 hpi does not interfere with the expression of the viral immediate early protein 1 (IE1), early protein pUL44, the late major capsid protein (MCP), or the viral protein kinase pUL97. Staining of β-actin served as an internal loading control. *DMSO*, dimethyl sulfoxide.
(TIF)

S8 Fig Localization of emerin is not affected by PiB treatment in uninfected and HCMV-infected cells. Primary human foreskin fibroblasts (HFFs) were infected with HCMV AD at a MOI of 0.01 or remained uninfected (mock). At 48 hpi, cells were treated with DMSO (dimethyl sulfoxide) or 10 μM PiB as indicated. Cells were fixed at 72 hpi followed by immunofluorescence staining using rabbit pAb-UL97 and mouse mAb-emerin. Cell nuclei were counterstained with DAPI (4’,6-diamidino-2-phenylindole). Samples were analysed by confocal microscopy. *Scale bars*, 10 μm.
(TIF)

S1 Dataset Intracellular intensities of Ser22-specific lamin A/C phosphorylation in herpesvirus-infected primary fibroblasts. Signal intensities were determined for infected and surrounding uninfected cells from maximum projections of confocal z-series for the indicated viruses. The first index card shows a summary of mean signal intensities and mean percentages of cells with increased lamin phosphorylation from three independent experiments for each virus. Note that the mean values ± standard deviation are also depicted in Table 2. The following index cards show the signal intensities of individual cells for each virus setting. P values were determined by Student’s t-test.
(XLSX)

S2 Dataset Intracellular intensities of Ser392-specific lamin A/C phosphorylation in herpesvirus-infected primary fibroblasts. Signal intensities were determined for infected and surrounding uninfected cells from maximum projections of confocal z-series for the indicated viruses. The first index card shows a summary of mean signal intensities and mean percentages of cells with increased lamin phosphorylation from three independent experiments for each virus. Note that Table 2 contains the mean values ± standard deviation of three independent experiments. The following index cards show the signal intensities of individual cells for each virus setting. P values were determined by Student’s t-test.
(XLSX)

S1 Table Oligonucleotides used in this study. **Oligonucleotides used for the generation of expression plasmids coding for lamin A point mutants.** Coding sequences are given in bold and substituted nucleotides for site-directed mutation are underlined and bold.
(DOCX)
