# Supplementary figures and images for: The Prolyl Isomerase Pin1 Promotes the Herpesvirus-Induced Phosphorylation-Dependent Disassembly of the Nuclear Lamina Required for Nucleocytoplasmic Egress
Source: PLoS Pathog. 2016 Aug 24;12(8):e1005825. doi: 10.1371/journal.ppat.1005825 (PMC4996521; doi:10.1371/journal.ppat.1005825)

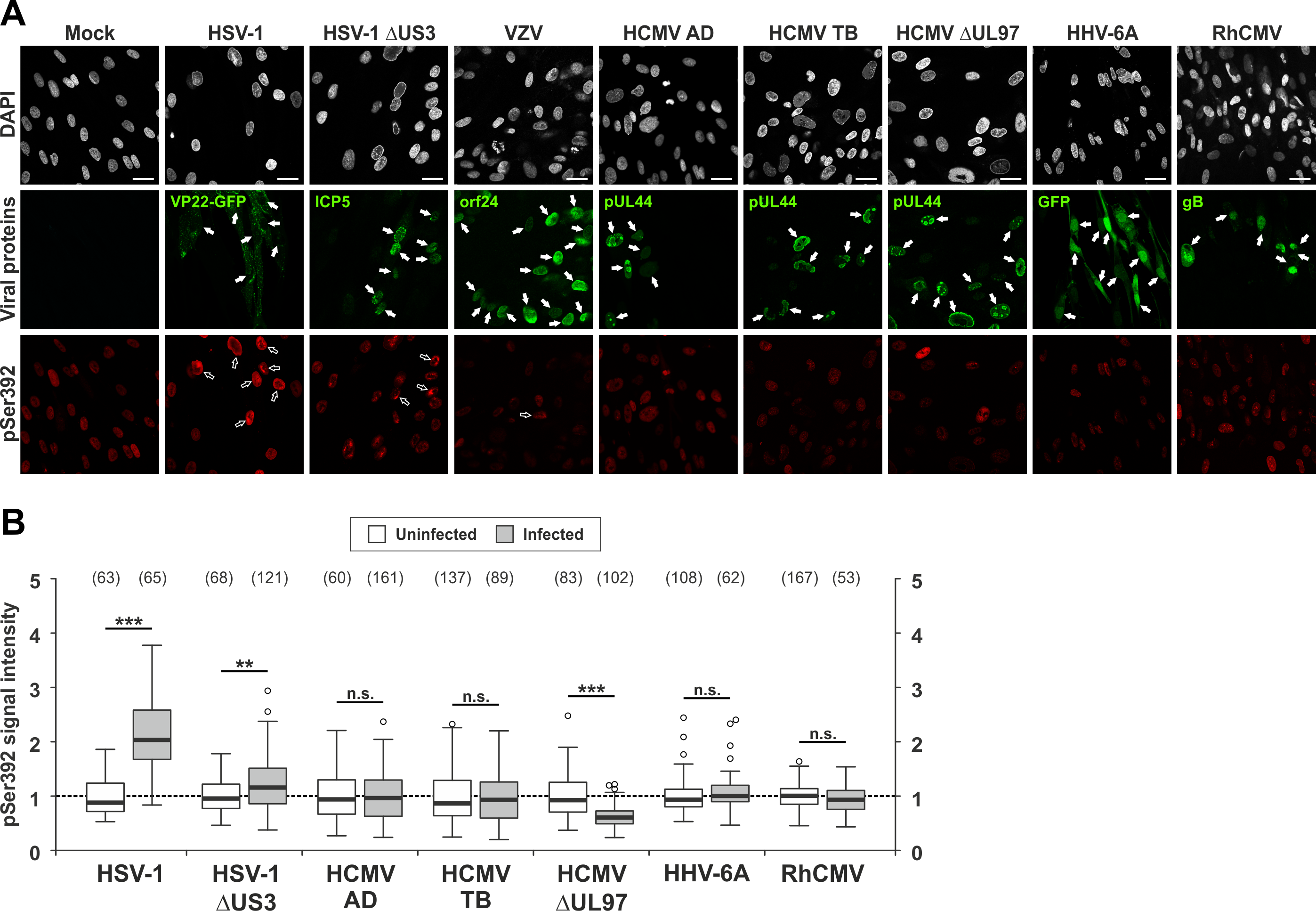

Supplement: S1 Fig — (A) HFFs were infected with different herpesviruses or remained uninfected (mock) as indicated. Cells were fixed at 24 hpi (HSV-1 and HSV-1 ΔUS3) or 72 hpi (VZV, HCMV AD, HCMV TB, HCMV ΔUL97, HHV-6A, and RhCMV) followed by immunofluorescence analysis using phospho-specific antibodies to detect lamin A/C phosphorylated at Ser392 in red. Staining of viral proteins or the green fluorescent protein (GFP) served as viral markers in green. Cell nuclei were counterstained with DAPI (4’,6-diamidino-2-phenylindole). Samples were analysed by confocal microscopy and a representative image of the focal plane is depicted for each setting. Filled arrows, nuclei of virus-positive cells; open arrows, nuclei of virus-positive cells showing increased Ser392 phosphorylation compared to virus-negative cells; scale bars, 30 μm. (B) Median intracellular intensities of lamin A/C phosphorylation. pSer392 signals were determined for infected (white boxes) and surrounding uninfected cells (grey-shaded boxes) as maximum projections of confocal z-series. One representative experiment out of three is depicted for each virus presenting the values of site-specific phosphorylation as box plots. Note, Table 2 contains the mean values ± standard deviation of three independent experiments. Centre lines show the medians with box limits indicating the 25th and 75th percentiles as determined by R software. Whiskers extend 1.5 times the interquartile range from the 25th and 75th percentiles, outliers are represented by circles, and the number of evaluated cells is depicted above each box in brackets. Statistical significance was determined by Student’s t-test (*, P < 0.05; **, P < 0.01; ***, P < 0.001; n.s., not significant, P ≥ 0.05). (TIF) [file ppat.1005825.s001.tif]

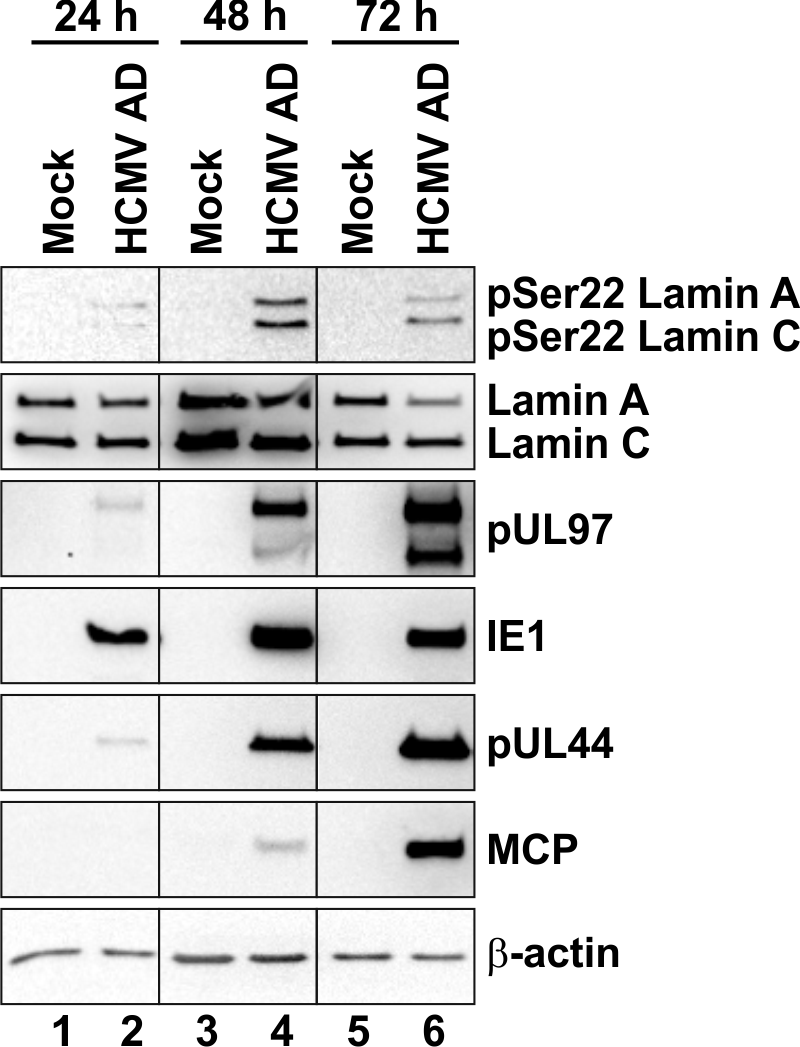

Supplement: S2 Fig — HFFs were infected with HCMV AD at a MOI of 1.0 or remained uninfected (mock). Cells were lysed at 24 hpi, 48 hpi, and 72 hpi. Total lysates were subjected to standard Western blot analysis for detection of lamin A/C phosphorylated at Ser22 (pSer22; upper panel), total lamin A/C (second panel), viral protein kinase pUL97 (third panel), viral immediate early protein 1 (IE1; fourth panel), viral early protein pUL44 (fifth panel), the viral late major capsid protein (MCP; sixth panel), and the loading control β-actin (lower panel). (TIF) [file ppat.1005825.s002.tif]

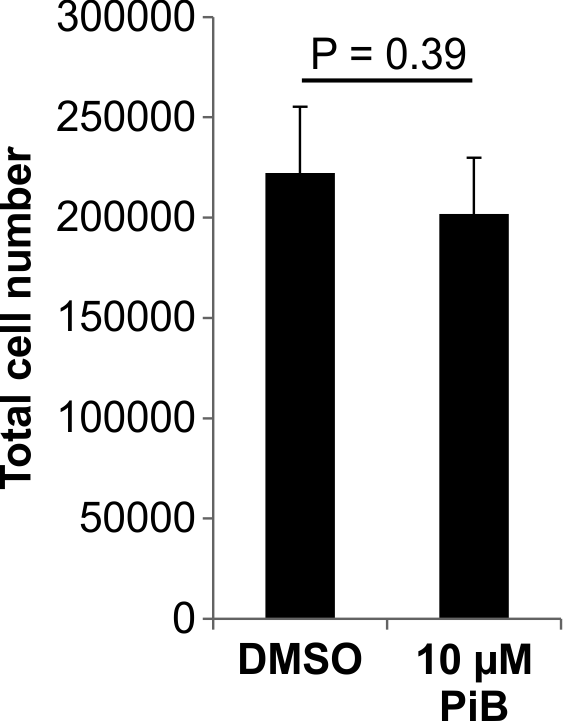

Supplement: S3 Fig — Primary human foreskin fibroblasts (HFFs) were treated with 10 μM PiB or dimethyl sulfoxide (DMSO) as solvent control. At 7 d post-treatment, cell proliferation was evaluated by microscopic counting of total cell numbers (n = 4). P values were determined by Student’s t-test. (TIF) [file ppat.1005825.s003.tif]

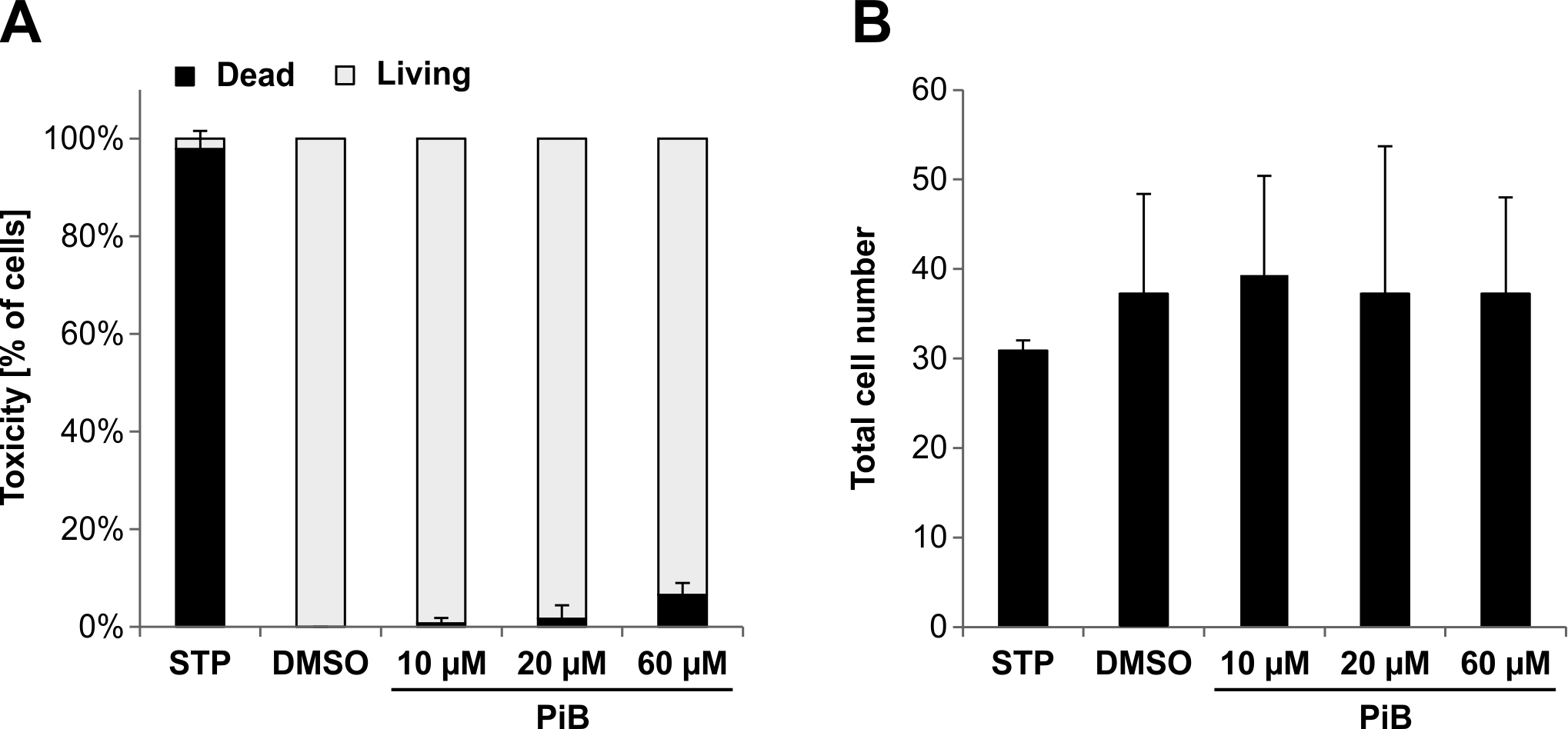

Supplement: S4 Fig — Primary human foreskin fibroblasts (HFFs) were treated with increasing amounts of PiB, dimethyl sulfoxide (DMSO) as solvent control, or staurosporine (STP) in a toxic concentration of 10 μM. At 24 h post-treatment, the cells were stained with trypan blue followed by microscopic counting of living and dead cells (n = 3). (A) Mean percentage of living (grey bars) and dead (black bars) cells. (B) Mean total cell numbers comprising dead and living cells. (TIF) [file ppat.1005825.s004.tif]

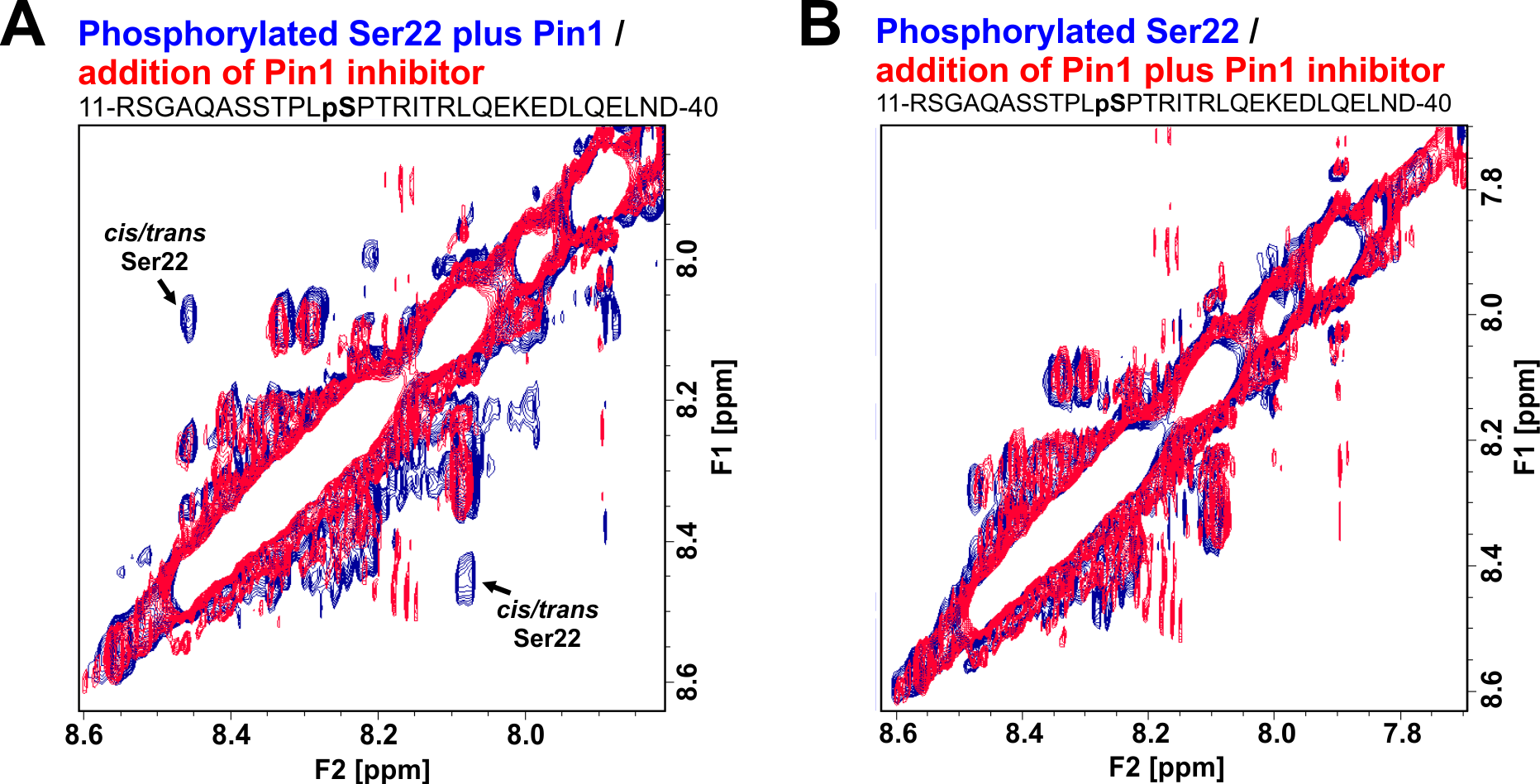

Supplement: S5 Fig — Superimposed expanded HN-HN regions of the 2D 1H-1H NOESY spectra are depicted for phosphorylated and unphosphorylated versions of a lamin A/C peptide comprising amino acids 11–40. (A) Phosphorylated peptide after addition of Pin1 (blue signals) and after additional treatment with the Pin1 inhibitor juglone (red signals); note that prolyl cis/trans related exchange peaks detected after addition of Pin1 disappear after treatment with juglone. (B) Phosphorylated peptide prior to (blue signals) and after addition of both Pin1 and the Pin1 inhibitor juglone (red signals); note that the spectrum recorded after addition of both Pin1 and the Pin1 inhibitor juglone resembles the spectrum of the pure peptide. (TIF) [file ppat.1005825.s005.tif]

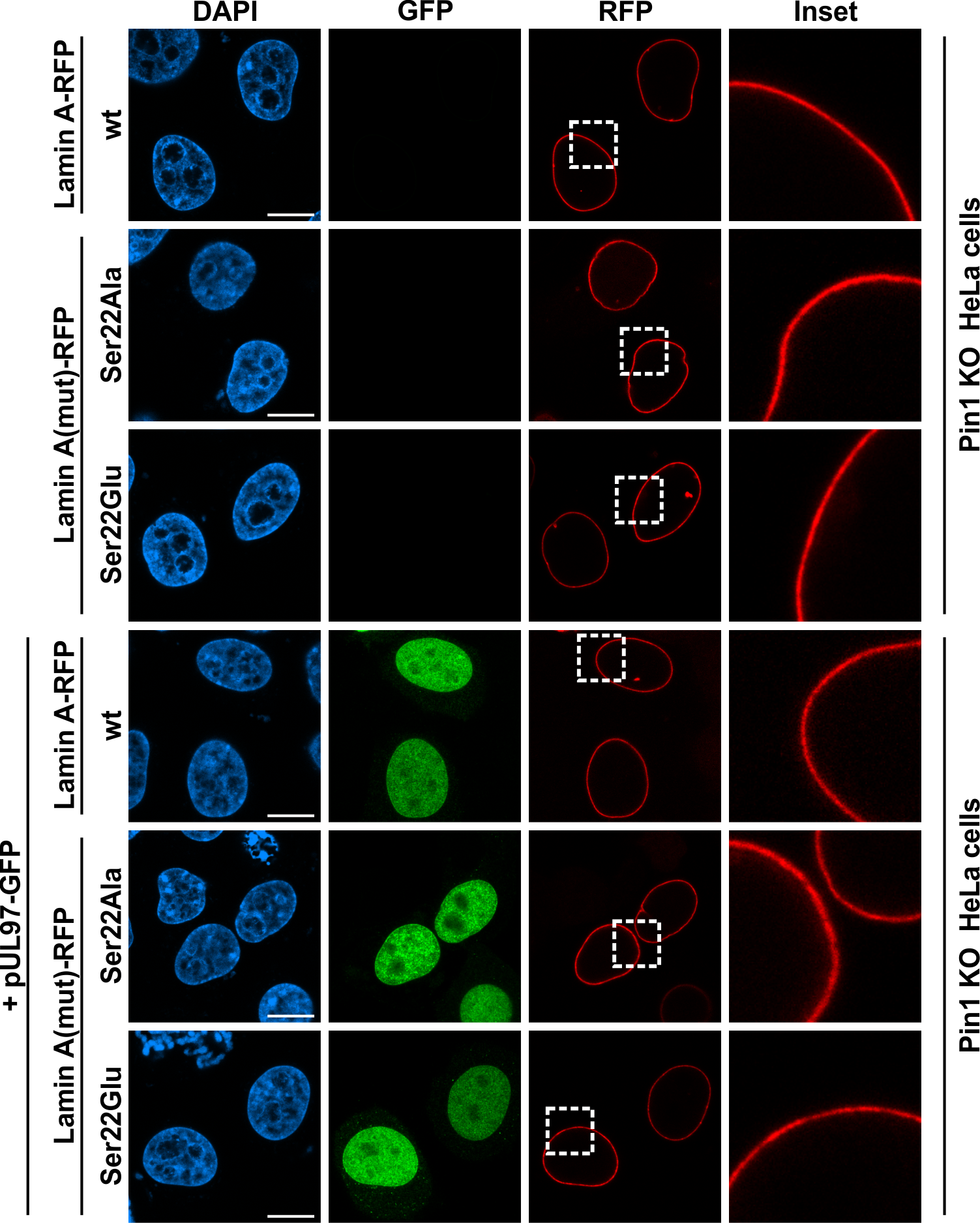

Supplement: S6 Fig — Pin1 knockout (KO) HeLa cells were transiently cotransfected with plasmids coding for HCMV pUL97 fused to the green fluorescent protein (GFP) and wild-type (wt) or mutant lamin A fused to the red fluorescent protein (RFP) as indicated. Cells were fixed at 24 h post-transfection followed by counterstaining of cell nuclei with DAPI (4’,6-diamidino-2-phenylindole). Samples were analysed by confocal microscopy. Insets show the magnification of dashed boxes. Scale bars, 10 μm. (TIF) [file ppat.1005825.s006.tif]

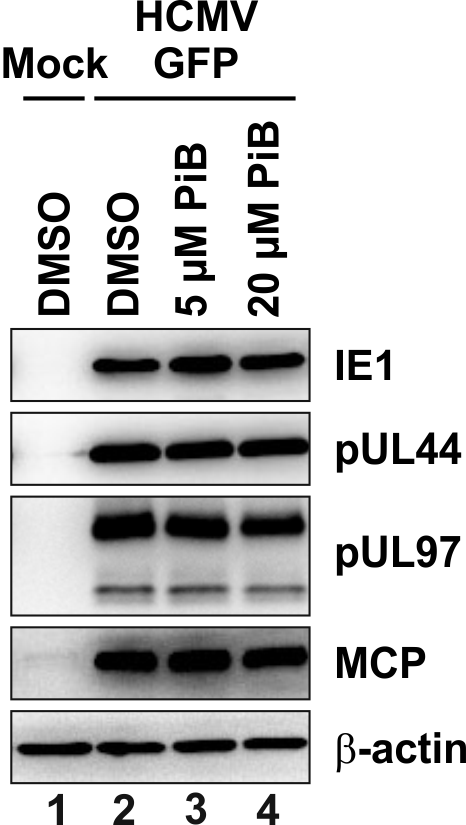

Supplement: S7 Fig — Primary human foreskin fibroblasts (HFFs) were infected with the human cytomegalovirus (HCMV) strain AD169-GFP and treated with the Pin1 inhibitor PiB added at 48 hpi at indicated concentrations of 5 or 20 μM. Western blot analysis was performed with total lysates taken at 72 hpi. Note that PiB addition at 48 hpi does not interfere with the expression of the viral immediate early protein 1 (IE1), early protein pUL44, the late major capsid protein (MCP), or the viral protein kinase pUL97. Staining of β-actin served as an internal loading control. DMSO, dimethyl sulfoxide. (TIF) [file ppat.1005825.s007.tif]

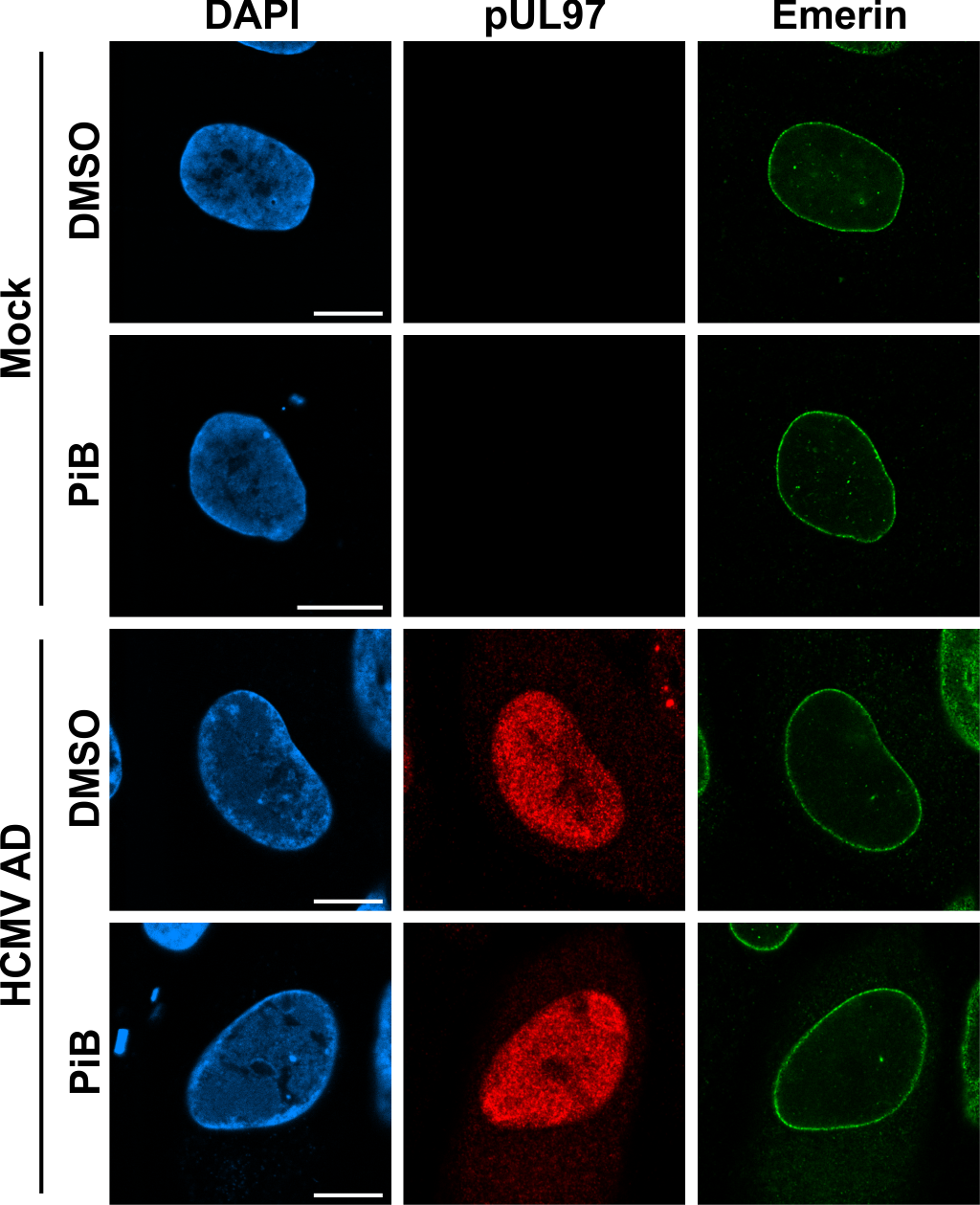

Supplement: S8 Fig — Primary human foreskin fibroblasts (HFFs) were infected with HCMV AD at a MOI of 0.01 or remained uninfected (mock). At 48 hpi, cells were treated with DMSO (dimethyl sulfoxide) or 10 μM PiB as indicated. Cells were fixed at 72 hpi followed by immunofluorescence staining using rabbit pAb-UL97 and mouse mAb-emerin. Cell nuclei were counterstained with DAPI (4’,6-diamidino-2-phenylindole). Samples were analysed by confocal microscopy. Scale bars, 10 μm. (TIF) [file ppat.1005825.s008.tif]
