# Supplementary material for: The Prolyl Isomerase Pin1 Promotes the Herpesvirus-Induced Phosphorylation-Dependent Disassembly of the Nuclear Lamina Required for Nucleocytoplasmic Egress
Source: PLoS Pathog. 2016 Aug 24;12(8):e1005825. doi: 10.1371/journal.ppat.1005825 (PMC4996521; doi:10.1371/journal.ppat.1005825)
Supplement: S1 Table — Oligonucleotides used for the generation of expression plasmids coding for lamin A point mutants. Coding sequences are given in bold and substituted nucleotides for site-directed mutation are underlined and bold. (DOCX) [file ppat.1005825.s011.docx]

**Table S1.** **Oligonucleotides used in this study.** **Oligonucleotides used for the generation of expression plasmids coding for lamin A point mutants.** Coding sequences are given in bold and substituted nucleotides for site-directed mutation are underlined and bold.

| **Primer** | **Sequence (5’-3’)** |
| --- | --- |
| 5-LamA(S22A) | **ccagctccactccgctgGcgcccacccgcatcacc** |
| 3-LamA(S22A) | **GGTGATGCGGGTGGGCGCCAGCGGAGTGGAGCTGG** |
| 5-LamA(S22E) | **ccagctccactccgctgGAgcccacccgcatcacc** |
| 3-LamA(S22E) | **GGTGATGCGGGTGGGCTCCAGCGGAGTGGAGCTGG** |
